# Fault reactivation and earthquakes with magnitudes of up to Mw4.7 induced by shale-gas hydraulic fracturing in Sichuan Basin, China

**DOI:** 10.1038/s41598-017-08557-y

**Published:** 2017-08-11

**Authors:** Xinglin Lei, Dongjian Huang, Jinrong Su, Guomao Jiang, Xiaolong Wang, Hui Wang, Xin Guo, Hong Fu

**Affiliations:** 1 Geological Survey of Japan, AIST, Tsukuba, 305-8567 Japan; 2 Yibin Earthquake Mitigation Administration, Yibin, 64400 China; 3 Earthquake Monitoring Centre, Sichuan Earthquake Administration, Chengdu, 610041 China; 4 Department of Science and Technology, Chongqing Earthquake Administration, Chongqing, 401137 China; 5 Center for Research and Prediction, Yunnan Earthquake Administration, Kunming, 650224 China

## Abstract

This paper presents a timely and detailed study of significant injection-induced seismicity recently observed in the Sichuan Basin, China, where shale-gas hydraulic fracturing has been initiated and the aggressive production of shale gas is planned for the coming years. Multiple lines of evidence, including an epidemic-type aftershock sequence model, relocated hypocenters, the mechanisms of 13 large events (*M*_*W*_ > 3.5), and numerically calculated Coulomb failure stress results, convincingly suggest that a series of earthquakes with moment magnitudes up to *M*_*W*_ 4.7 has been induced by “short-term” (several months at a single well pad) injections for hydraulic fracturing at depths of 2.3 to 3 km. This, in turn, supports the hypothesis that they represent examples of injection-induced fault reactivation. The geologic reasons why earthquake magnitudes associated with hydraulic fracturing operations are so high in this area are discussed. Because hydraulic fracturing operations are on the rise in the Sichuan Basin, it would be beneficial for the geoscience, gas operator, regulator, and academic communities to work collectively to elucidate the local factors governing the high level of injection-induced seismicity, with the ultimate goal of ensuring that shale gas fracking can be carried out effectively and safely.

## Introduction

When any type of fluid is pressure-injected into an underground reservoir, as is done during fluid waste disposal and shale gas hydraulic fracturing (also referred to as hydro-fracturing or fracking), the pressure of the fluids (pore pressure) underground increases, and the underground stress distribution may change. Stress redistribution and pore pressure changes within and surrounding the reservoir may lead to geomechanical changes, fault reactivation, micro-seismicity, and even damaging earthquakes^1–7^. The abnormal increase in seismicity in areas such as the central United States^8, 9^ and western Canada^10, 11^ is considered to be a direct result of the rapid increase in injections for several applications including wastewater disposal, enhanced oil recovery (EOR), and shale gas hydraulic fracturing. Such injection-induced seismicity appears to be strongly localized, and moment magnitude (*M*_*W*_) ≥ 3 events have only been directly tied with wells at a very limited number of sites^3, 12, 13^.

In recent years, shale gas production has grown rapidly because of the use of horizontal drilling and advancements in hydraulic fracturing technology. Simultaneously, earthquakes caused by the injections used for hydro-fracturing have become a point of significant worldwide public interest^14^, and a recent study has shown that “short-term” injections for hydraulic fracturing have probably induced earthquakes up to *M*_*W*_ ≥ 3 at some sites^13^. Shallow earthquakes of a moderate size (*M*_*W*_ 3–5) can be destructive^3^. In addition, the reactivation of pre-existing faults may hinder hydraulic fracturing and lead to the migration of injected fluid into shallower or deeper formations. Therefore, because the technological developments that address such public concerns are essential to the safe conduct of hydraulic fracturing, well operators must develop or be provided with tools that will allow them to manage or forecast the occurrence of destructive earthquakes, and to avoid the reactivation of pre-existing faults, especially those that connect shallower formations.

**Figure 1:**
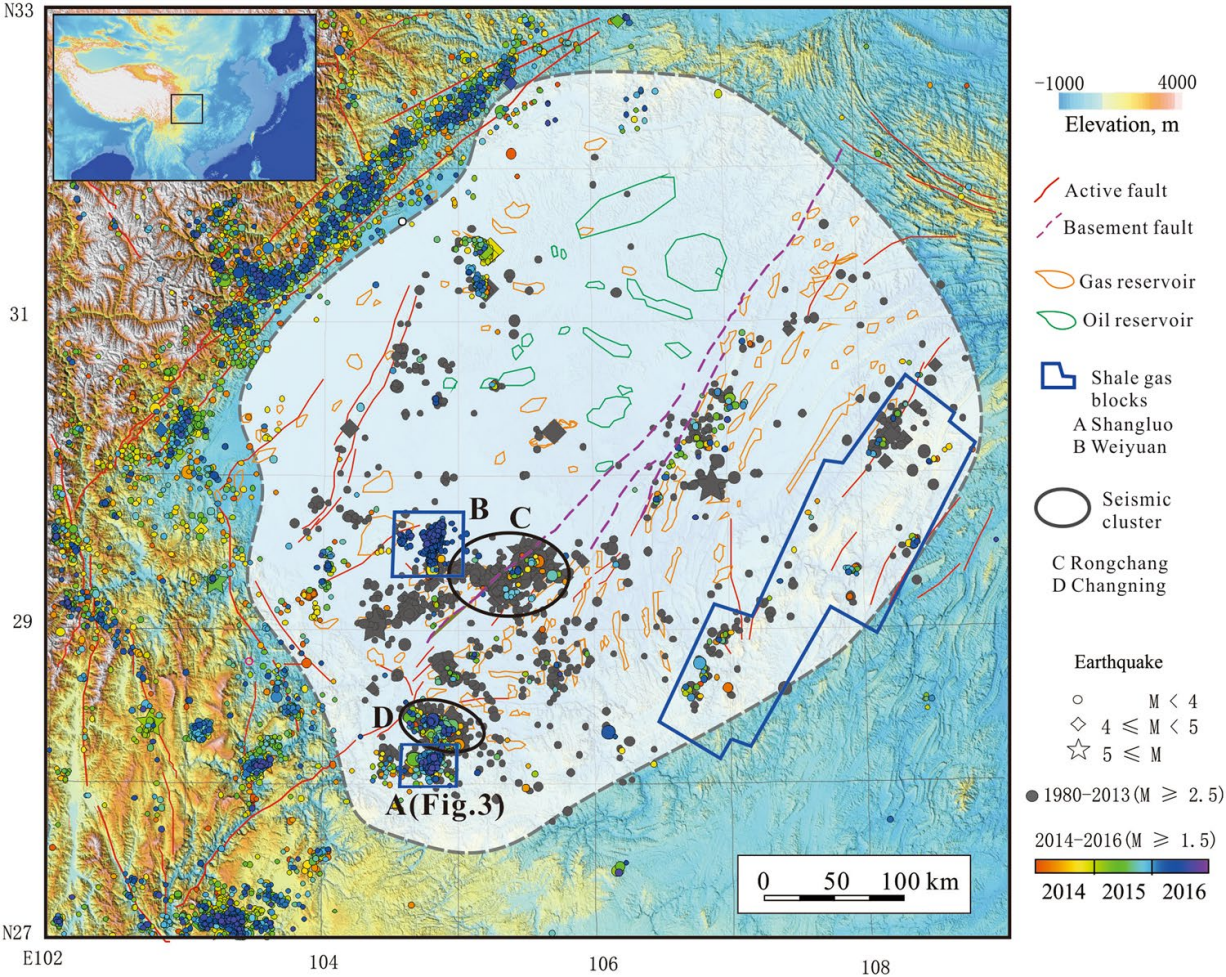
Seismicity of the Sichuan Basin and surrounding regions. The gray and colored dots denote seismic events with magnitudes of at least *M*_*L*_ 2.5 and *M*_*L*_ 1.5 that have occurred since 1980 and 2014, respectively. Fault data are imported from the digital “Map of active tectonics in China”^52^. The basement faults are hidden deep faults in the crystal basement and show minor activity^53^. The oil and gas field outlines are digitized and modified from an open report^54^. The Shangluo (‘A’, study area of this paper), Weiyuan (‘B’), and other major shale gas blocks in which hydraulic fracturing is in operation are also shown. Area ‘C’ covers the region of wastewater injection induced seismicity in gas-depleted reservoirs^3, 20^, while seismicity in area ‘D’ was induced by water injection in deep salt wells. The background topography is based on the Shuttle Radar Topography Mission (SRTM3) digital elevation model (DEM) data (http://srtm.csi.cgiar.org/SRTM3). The map was created using the free software GeoTaos_map (developed by Xinglin Lei; https://staff.aist.go.jp/xinglin-lei/) and finished with the software CorelDRAW × 8. (Copyright (**c**) 2016 Corel Corporation. All rights reserved.)

Herein, we focus on shale gas site blocks in the town of Shangluo and its environs in the Sichuan Basin, China (indicated as ‘A’ in Fig. 1 and Supplementary Fig. 1), referred to hereafter as “the study area”. So far, five *M*_*W*_ > 4.0 earthquakes have occurred concurrently with fracturing operations. The largest of these (*M*_*W*_ 4.7), which occurred on 28 Jan. 2017, caused significant damages to nearby farmhouses and other constructions (Supplementary Fig. 21). This event was probably the largest earthquake induced by fracking injections reported so far. The remainder of the present paper is organized as follows. First, we briefly describe the seismicity and hydraulic fracturing operations in the study area. Then, we present the results of this study, including statistical analyses, moment tensor inversion of the largest earthquakes (*M*_*W*_ > 3.5), hypocenter relocation, numerically calculated Coulomb Failure Stress results, and a seismogenic index. These are followed by a discussion and conclusion section. Finally, the Methods section is presented to explain the summarizing methods used in this study.

## Seismicity and hydraulic fracturing operation in Shangluo shale gas site

In the Shangluo shale gas site (indicated as ‘A’ in Fig. 1 and Supplementary Fig. 1), the target reservoir for hydraulic fracturing operations is a Silurian mudstone/shale formation that has a burial depth of approximately 2.3 to 3 km. This site corresponds to a wide and flat syncline. In the period from 1970 to Oct. 2008, only 60 earthquakes with *M*_*L*_ greater than 2.5 were observed within the study area (Supplementary Fig. 4), indicating that the area has a low level of background seismicity. However, between the start of shale gas prospecting in 2008 and 2012, an increasing event rate was observed (Supplementary Fig. 4a). Based on our survey, we believed that the limited well injections conducted for evaluation purposes might be responsible for the increase (Supplementary Fig. 4). Horizontal drilling began in 2011, while systematic shale gas hydraulic fracturing in those horizontal wells began in 2014. Since Dec. 2014, the earthquake rate dramatically increased (Supplementary Fig. 4b) in limited areas surrounding the hydraulic fracturing well pads, where (based on interviews during field surveys) local inhabitants reported feeling quakes at an abnormally high frequency. No conventional gas/oil reservoirs have been found within the study area. Thus far, wastewater, including coproduced water with gas production and hydraulic fracturing flowback water, was transported and injected into two disposal wells, located in places outside of the study area^15^. So far, all injections in the study area are for shale fracking purposes.

In the study area, well pads intended for hydraulic fracturing generally have four to eight wellbores with horizontal lengths up to approximately 2500 m (Supplementary Fig. 3). The interval between the lateral portions of two neighbor wells is generally 300 to 400 m^16^. The lateral portions are commonly drilled in the direction parallel to hydraulic fracture opening (perpendicular to the planes of the fractures), which is perpendicular to the maximum horizontal principal stress, in order to maximize the volume stimulated by the induced fractures. In our study area, borehole breakout data consistently indicate that the maximum horizontal stress axis trends approximately from N100°E to N115°E^17, 18^ (Supplementary Fig. 3). A multistage hydraulic fracturing technique was applied for treatment. Both single- and multi-well completions are used^16^. In the multi-well completions, the so-called zipper fracturing technique, which involves staggered injection stages between two or three wells, was commonly applied^16^. On average, more than 1800 m^3^ water is required during a single stage of hydraulic fracturing^15, 16^. The average injection rate (approximately 11–12 m^3^/min), average injected fluid volume, and average surface pumping pressure (approximately 60–70 MPa) are similar for all stages and all wells^15, 16^. For a single well pad of six horizontal wells, a total water volume of more than 150,000 m^3^ is required, and amounts can reach 200,000 m^3^ in some cases. More than 2 months of fracturing are required for a typical pad of six wellbores^16^.

**Figure 2:**
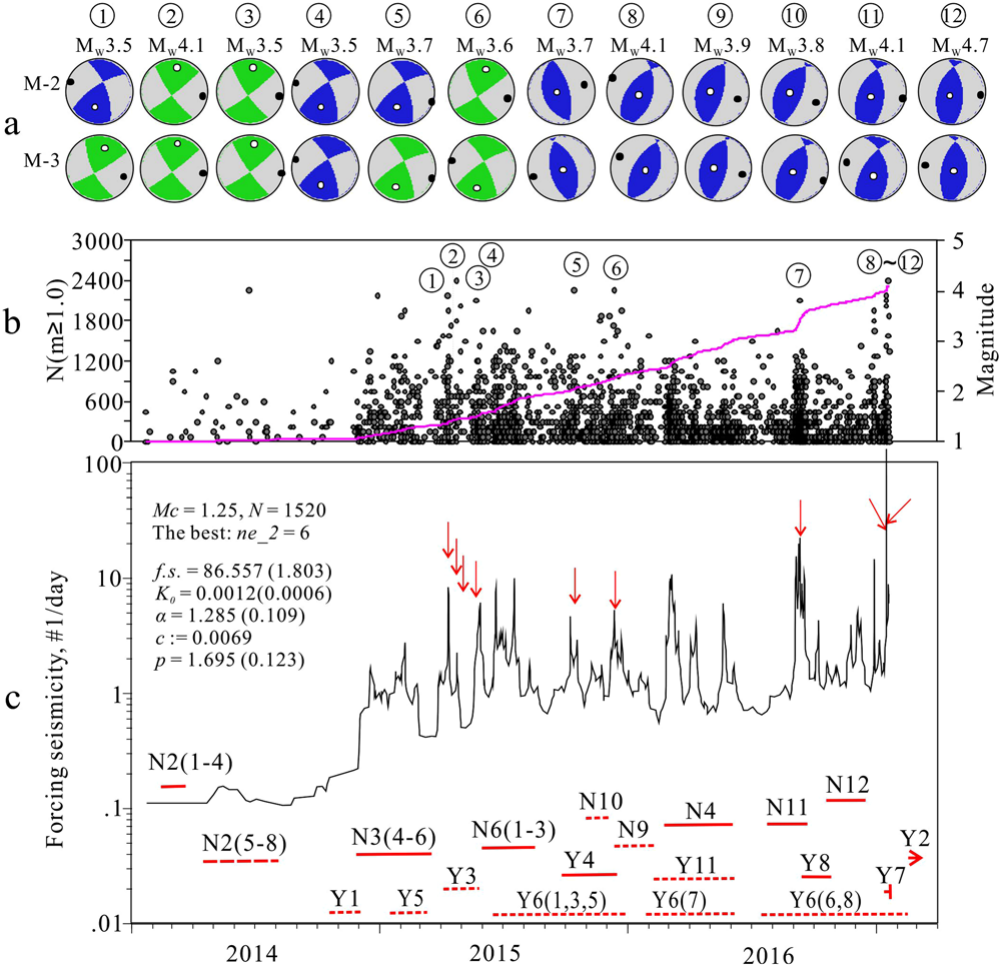
Epidemic-type aftershock sequence (ETAS) model results for earthquakes occurred in the Shangluo shale gas block (Fig. 1A) from 2014 to 20 Jan. 2017. (**a**) Mechanism solutions of the largest earthquakes (indicated by circled number and arrows) estimated by the gCAP method using two velocity models. (**b**) M-t and N-t plots. (**c**) ETAS parameters and their standard errors (number in brackets) (see Method section and Supplementary Fig. 5 for details). The time-varying forcing rate is plotted with respect to time. The red solid and dashed lines show major hydraulic fracturing time windows, and N# and Y# indicate the associated well pad numbers in the Ning-201 and Ys108 shale blocks, respectively.

Coinciding with the start of systematic horizontal well shale gas hydraulic fracturing, the observed earthquake rate within the study area increased dramatically (Fig. 2b). During the period from Dec. 2014 to 20, Feb. 2017, more than 2,400 *M*_*L*_ ≥ 1.0 events were observed there, including four *M*_*W*_ ≥ 4.0 events. The largest *M*_*W*_ 4.7 event heavily damaged structures in the nearest villages (23 houses collapsed and 548 houses were heavily damaged) (Supplementary Fig. 21). Three factors might be responsible for making this event particularly destructive: (1) a very shallow focal depth of only 1.8 km (Supplementary Fig. 14); (2) a reverse mechanism (Supplementary Fig. 14); and (3) the fact that two villages were located within 2 km of the epicenter (Supplementary Fig. 21). It should be noted that another large event occurred on 4 May 2017 having a *M*_*W*_ of 4.6, focal depth of 2.5 km, and strike-slip mechanism solution (Supplementary Fig. 17), but was not so destructive. Comparisons of magnitude versus time (M-t) plots for earthquakes and major hydraulic fracturing operations clearly show that significant fluctuations in the seismic event rate correspond with the timing of nearby hydraulic fracturing operations (Supplementary Fig. 4a,b). The frequency-magnitude distribution shows a magnitude of completeness of 1.25 and a *b*-value of approximately 0.9 (Supplementary Fig. 4c). Note that a *b*-value close to 1.0 is a sign of fault activation (injection induced or not), but much higher microseismicity values directly caused by fractures opening during hydraulic fracturing operations are commonly observed^19^.

## Characteristics of fracking-induced earthquakes

To examine the statistical features of the observed seismicity, we applied our seismic dataset to the epidemic-type aftershock sequence (ETAS) model, which is useful for statistically separating the total seismicity into external forced background activity and Omori-type aftershocks^3, 20–22^. When used with a time-varying forcing rate, the ETAS model results show that the seismicity is governed by an external force (here, injection-related stress) with a total forcing rate of up to 87% and that Omori-type aftershocks are rare, encompassing only 13% of all earthquakes (Fig. 2). These results show similarities with recorded seismicity induced by injection of wastewater into depleted conventional gas reservoirs at other sites^3, 20^. In fact, the largest earthquakes in the investigated series did not produce significant aftershocks, as could be expected for tectonic earthquakes. In general, the seismicity rate decreased to a much lower level quickly after hydraulic fracturing ceased. However, in some cases, persistent seismicity continued for several tens of days after completion (Fig. 2).

**Figure 3:**
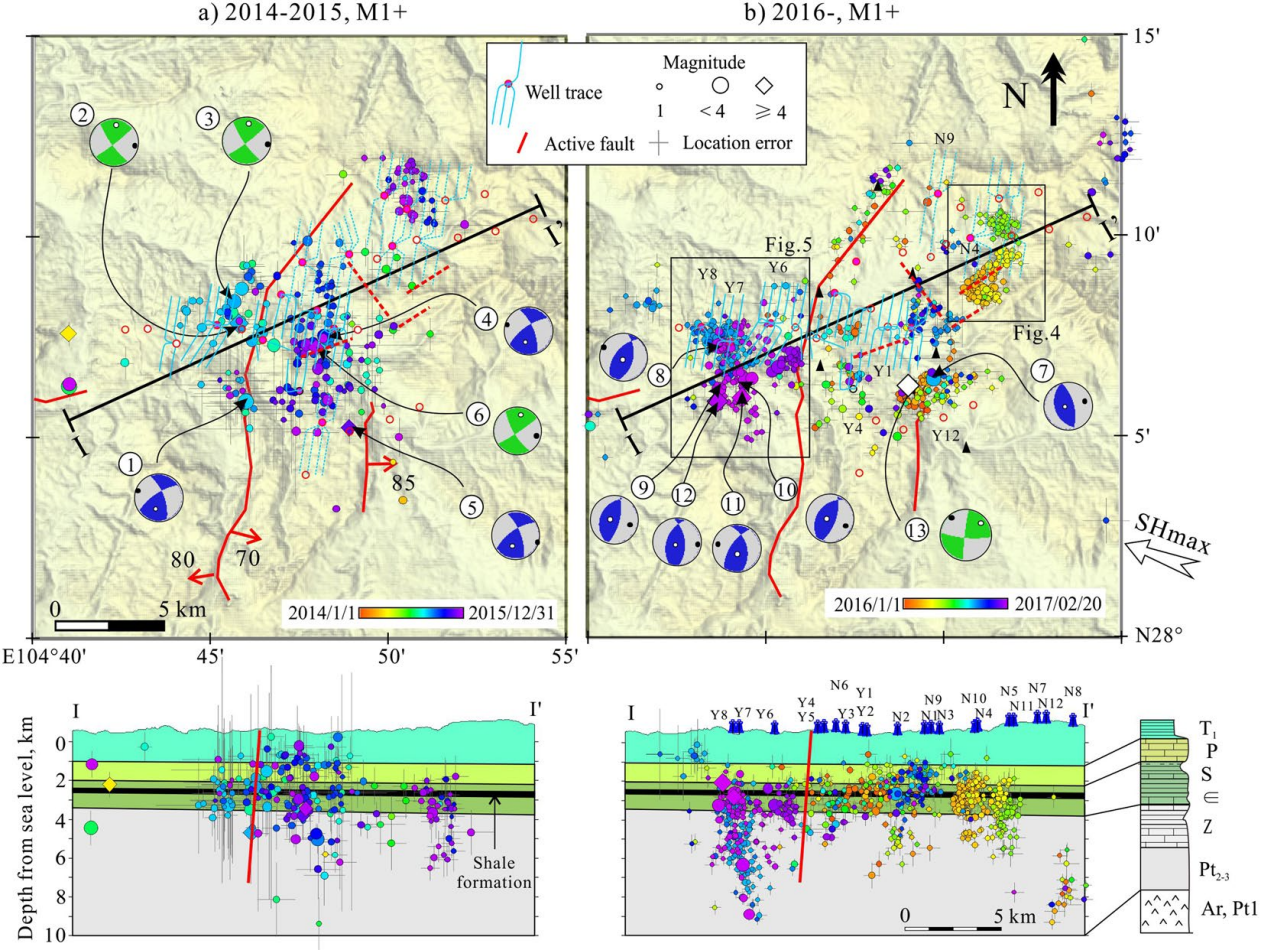
Map and section views of relocated earthquakes for two periods: (**a**) Jan. 1, 2014 through 2015, and (**b**) since Jan. 1, 2016, with the focal mechanisms of the largest earthquakes (*M*_*w*_ ≥ 3.5). The earthquake symbols are colored by date and scaled for magnitude. The gray cross lines show vertical and the horizontal errors given by the relocation program. The red arrows and numbers on the faults indicate the dip direction and dip angle of the fault. The big inward-pointed arrow shows the orientation of the maximum horizontal principal stress (SHmax). This map was created using the free software GeoTaos_map (developed by Xinglin Lei; https://staff.aist.go.jp/xinglin-lei/) and finished with the software CorelDRAW × 8. (Copyright (**c**) 2016 Corel Corporation. All rights reserved.)

Mechanism solutions of the 13 largest earthquakes (*M*_*W*_ > 3.5), based on moment tensor inversion using the generalized cut and paste method (gCAP)^23^, showed very consistent results (Table 1; Figs 2a and 3), with the best focal depth, corresponding to the minimum misfit error, falling in the range from 1.8 to 4 km (Table 1). Most events showed nearly pure double-couple (DC) mechanisms with very limited non-DC components (less than 4%), and only two events had a negative isotropic (ISO) component greater than 10%, probably due to uncertainty (Table 1). Two events showed strike-slip motion dominated mechanisms, three events showed strike-slip motion with a significant reverse component, and five events showed pure reverse faulting motion. Other earthquakes produced a reverse faulting mechanism with a lesser strike-slip component. The azimuth of the P-axes of all solutions, which were concentrated in a narrow azimuth range from 90° to 130° (Fig. 2a), agreed very well with the regional maximum horizontal stress (*σ*_*H*_) direction, which trends approximately from N100°E to N115°E as was previously mentioned. The fact that both strike-slip and reverse faulting earthquake were observed suggests that the regional minimum horizontal stress (*σ*_*h*_) is close to the vertical stress *σ*_*V*_.

**Table 1:** Mechanism solution of the largest earthquakes (*M*_*W*_ > 3.5).

#	EQ	FM1	FM2	*G*_*iso*_, *G*_*vlcd*_	*M*_*W*_	*M*_*L*_	*H*, km	H*, km
1	2015/04/11	339/63/26	237/67/150	−0.04, −0.00	3.54	3.9	3.3	2.6 ± 2.5
2	2015/04/25	144/76/71	52/83/166	+0.02, +0.00	4.05	4.2	2.96	5.1 ± 0.9
3	2015/04/30	145/76/9	53/81/166	−0.00, −0.01	3.51	3.7	2.9	2.7 ± 0.8
4	2015/05/23	−22/64/27	235/66/151	−0.12, −0.01	3.52	3.8	4.0	5.4 ± 0.8
5	2015/10/14	339/65/29	236/64/152	+0.04, +0.00	3.72	4.0	2.7	4.2 ± 1.2
6	2015/12/12	149/70/7	57/83/160	+0.07, +0.00	3.59	4.0	2.5	3.8 ± 0.6
7	2016/09/10	−14/60/93	160/30/85	−0.2, −0.03	3.66	3.8	3.4	2.94 ± 0.3
8	2017/01/15	15/42/74	216/50/104	0.04, 0.02	4.07	4.3	2.4	3.07 ± 0.5
9	2017/01/15	14/64/85	205/26/100	0.00, 0.00	3.86	3.7	2.7	3.70 ± 0.6
10	2017/01/15	19/61/82	216/30/105	0.00, 0.00	3.84	3.8	2.4	6.7 ± 0.4
11	2017/01/18	355/49/65	210/47/116	0.00, 0.00	4.11	4.3	1.8	3.3 ± 0.4
12	2017/01/28	−6/51/73	200/42/110	0.04, 0.02	4.67	4.9	1.8	5.1 ± 0.1
13	2017/05/04	186/84/14	95/76/174	−0.04, −0.04	4.56	4.9	2.4	

Columns FM1 and FM2 show strike/dip/rake of the two nodal planes. ***G***_***iso***_, ***G***_***vlcd***_ are respectively the squared ratios of the scalar potency of the ISO and CLVD component to the total scalar potency, representing their relative strengths^23^. *H* and *M*_*W*_ are central moment depth and moment magnitude of the best solution, respectively. For comparison, *M*_*L*_ from the catalog and focal depth (H*) and its standard error determined by the double differential relocation method are also shown. The focal depths are related to a mean surface level. A preferred velocity model (Supplementary Fig. 6 M-2) based on results of seismic ambient noise tomography of the Sichuan Basin was used for moment tensor inversion. From this table, we obtained an empirical relation between *M*_*L*_ of the catalog and *M*_*W*_ of the inversion: *M*_*W*_ = *M*_*L*_ − 0.22 (R^2^ = 0.85).

**Figure 4:**
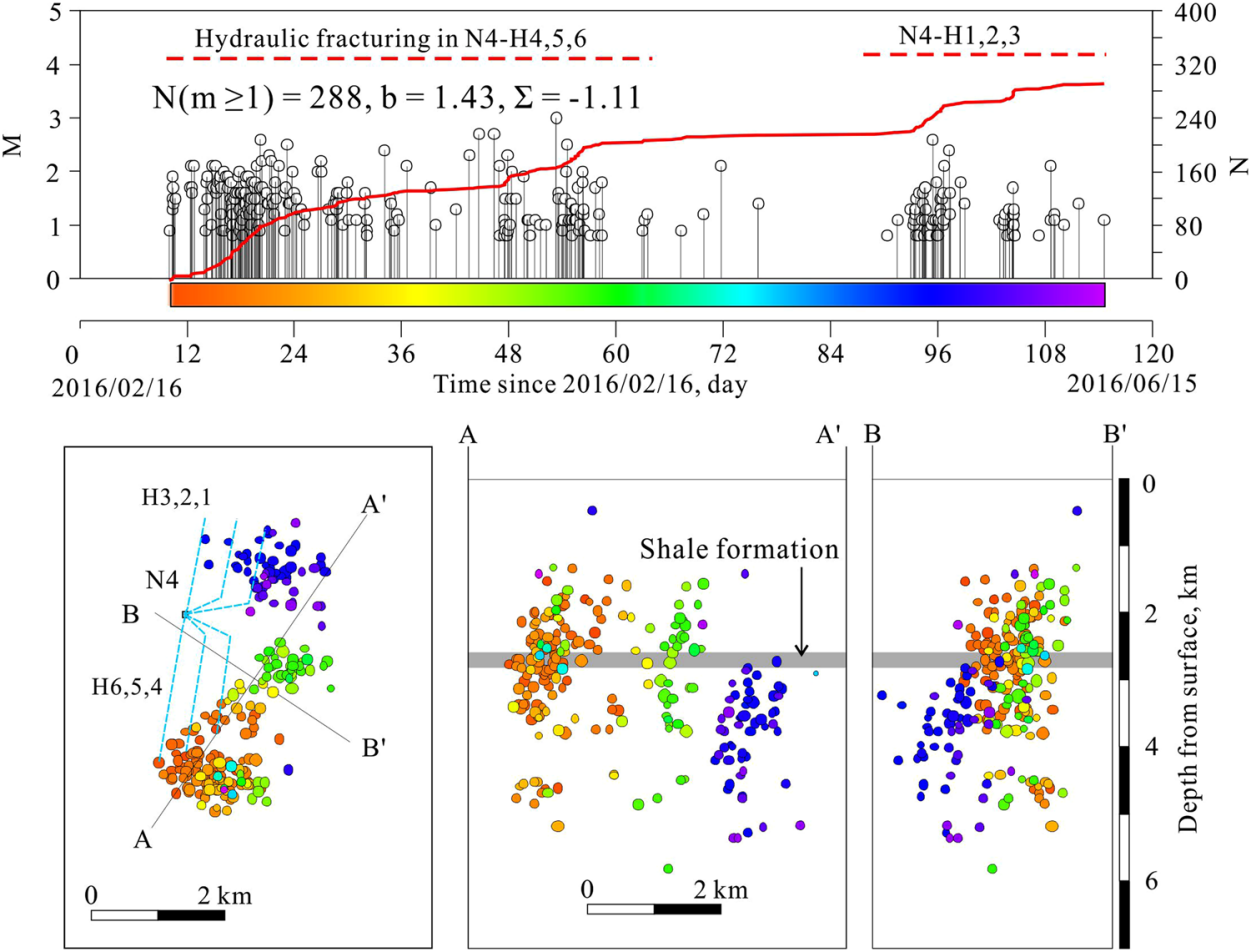
Detailed view of seismic cluster associated with well pad N4. The earthquake hypocenters are colored by date. Injection time window for well pad N4 is shown for comparison. The upper plot shows magnitudes and cumulative events, while the lower plots show map and vertical section views of earthquake distributions after double difference relocation. The calculated seismogenic index (Σ) is −1.11. The blue dashed lines in the map plot indicate traces of horizontal wells used for hydraulic fracturing treatment.

**Figure 5:**
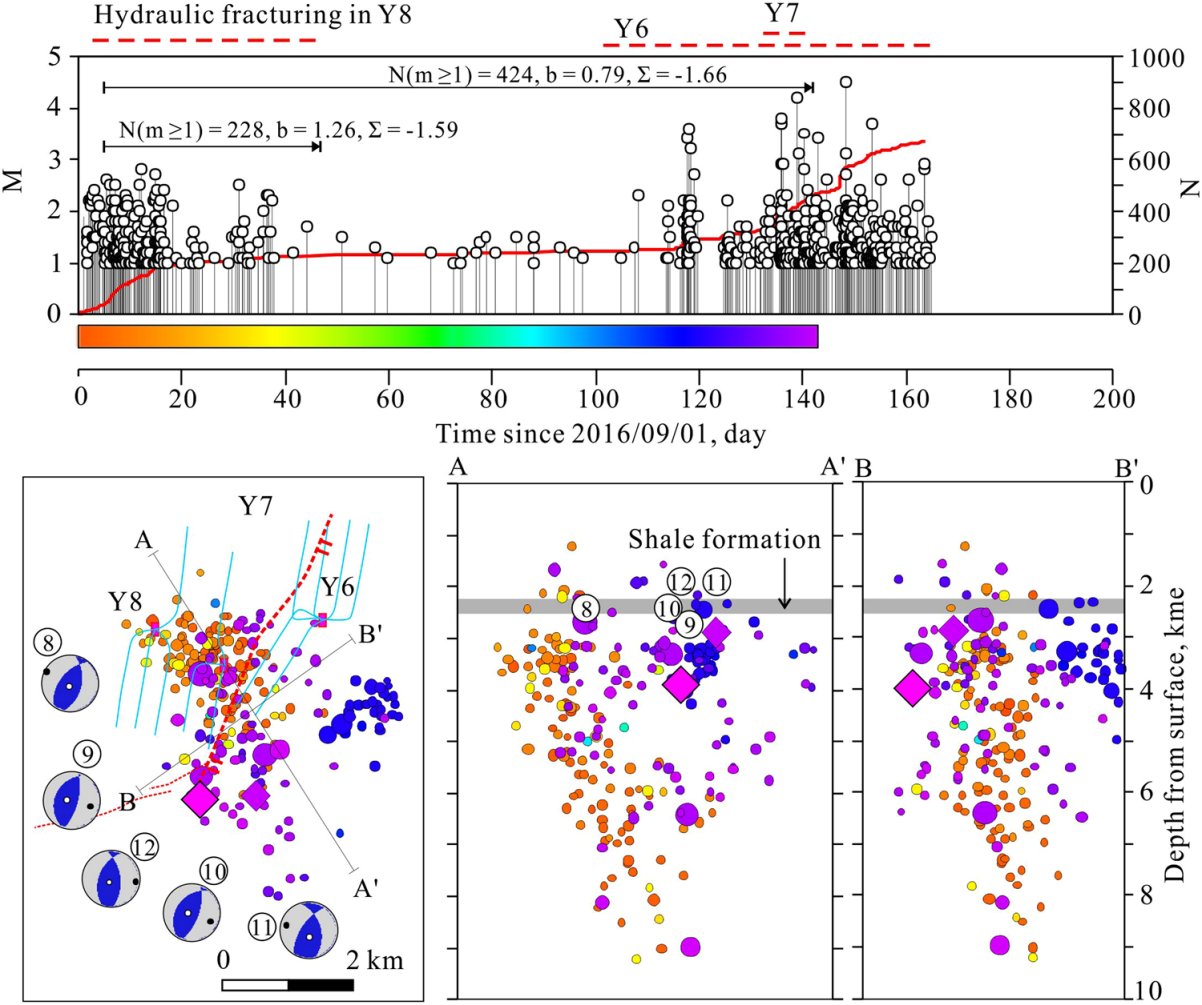
Detailed view of seismic cluster associated with well pads Y6, Y7, and Y8. The earthquake hypocenters are colored by date, and injection time windows for well pads Y6, Y7, and Y8 are shown for comparison. The upper plot shows magnitudes and cumulative events, while the lower plots show map and vertical section views of earthquake distributions after double difference relocation. The calculated seismogenic index (Σ) is −1.59. In the map view, red dashed lines indicate traces (at a depth of the shale formation) of major reverse faults, while the blue dashed lines indicate traces of horizontal wells used for hydraulic fracturing treatment. In section A-A’, the central moment depths are also shown.

The routinely determined earthquake hypocenters (reported by the Yibin Earthquake Mitigation Administration (YEB) and compiled in the China Earthquake Data Center (CEDC) catalog) are scattered around the well pads associated with the hydraulic fracturing operations (Supplementary Fig. 1b). Using phase data manually picked and compiled by the YEB and the Hypocenter Double-Difference (HypoDD) program^24^, we relocated a small number of earthquake hypocenters (208 of 1,045 events) for the period from 2014 through 2015. The resulting hypocenter locations showed definite improvements but remained imprecise, with estimated errors of up to a few kilometers (Fig. 3a). Assisted by the installation of six portable seismic stations at the end of 2015, 1,540 of the 3,666 earthquakes observed since 1 Jan. 2016 were resolved with horizontal errors less than a few hundred meters (Fig. 3b). Depth distributions of the relocated hypocenters showed a peak at depths of 3 to 4 km, with most events located in the upper parts of the underlying formations beneath the shale formation, which contains the producing interval that was being hydrofracked (Figs 3–5, Supplementary Fig. 18).

**Figure 6:**
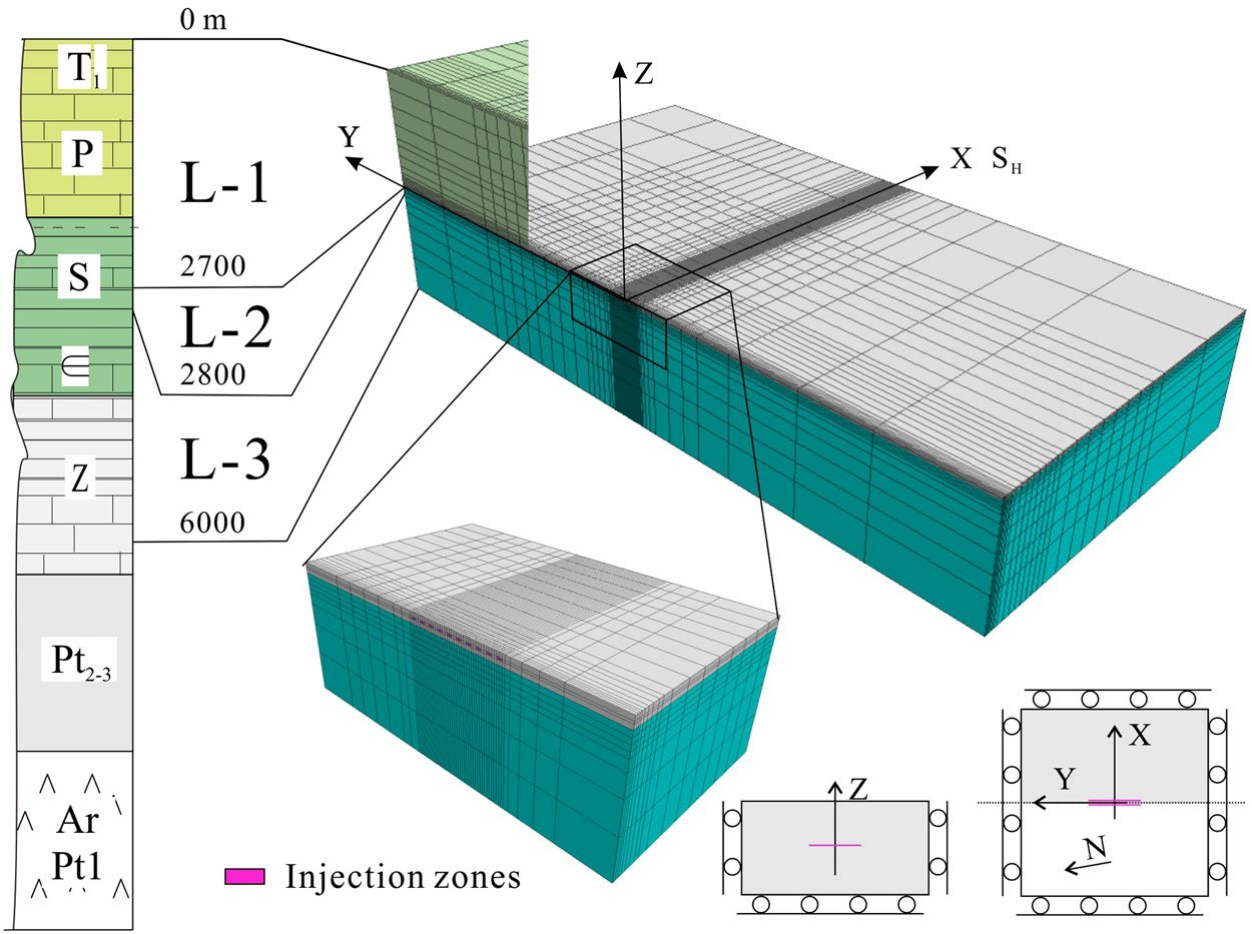
Numerical model for coupled thermal-hydrological-mechanical simulation of injections in horizontal wells for hydraulic fracturing.

**Figure 7:**
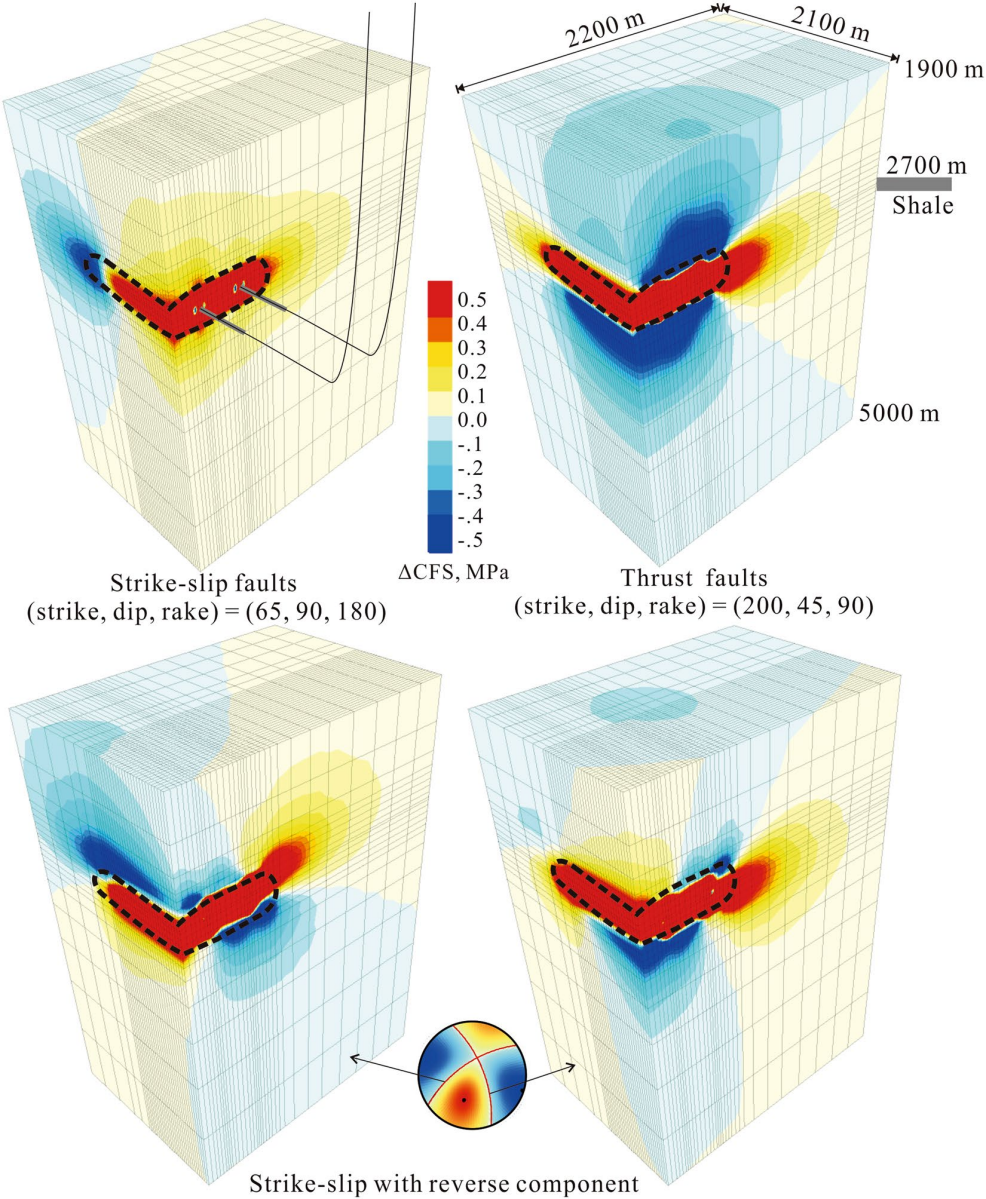
Estimated distributions of ΔCFS on favorably oriented strike-slip and thrust faults, and specified faults after 10-stage zipper fracturing in two wells. The dashed lines mark regions in which pore pressure and the total ΔCFS increased significantly.

The stress threshold for triggering earthquake faulting has been estimated to range from 0.01 to 0.05 MPa^25, 26^. A Coulomb failure stress change (ΔCFS) of a magnitude greater than 0.1 MPa is thus significant for critically stressed faults. Our coupled thermal-hydraulic-mechanic (THM) analysis, which is based on a simplified three-dimensional (3D) model (Fig. 6), indicates that after multistage treatments, significant ΔCFS may extend for ~2 km due to solid deformations (Fig. 7). If there are no permeable fault zones involved in the model, pore pressure diffusion is limited to within a distance of approximately 500 m, which is in agreement with the fact that microseismic activities directly induced by hydraulic fracture opening showed a spatial distribution within approximately 500 m of the fracturing intervals^27–29^. Since relocated hypocenter data indicate that the induced seismicity was scattered within in area of up to 2–3 km from the injection stages, and that persistent seismicity (although at a much lower rate) was observed between completion intervals (Figs 4 and 5), both solid deformation and pressure diffusion may have roles in inducing seismicity in our study area, in agreement with that observed in western Canada^13^.

Since our event catalog contains two temporally and spatially isolated earthquake clusters surrounding the N4 and Y7 pads (Fig. 3b), it is possible to show detailed analyses of events in these clusters and their associated hydraulic fracturing operations. The N4 pad includes three wells drilled southward from the pad and three wells drilled northward. Zipper fracturing in the southward wells began in Feb. 2016 and continued for 2 months. A *M*_*W*_ 3 event occurred in late stage of treatment. Zipper fracturing in the northward wells began in May 2016 and continued for 1 month (Fig. 4). The earthquake hypocenter distribution agrees fairly well with the ΔCFS pattern estimated for favorably oriented strike-slip faults (Fig. 7 and Supplementary Fig. 20).

Injections in the Y6, Y8, and Y7 pads were responsible for earthquakes surrounding the Y7 pad (Fig. 5). Treatments in the Y7 and Y6 pads are ongoing, and a *M*_*W*_ 4.7 event (the largest observed so far) occurred in the early morning of 28 Jan. 2017, causing significant damage to nearby farmhouses, as was previously mentioned (Supplementary Fig. 21). Before the largest event, four *M*_*W*_ = 3.7–4.3 events occurred in the period between 15 and 18 Jan. 2017 (Table 1) as the southward wells received treatment, which started in 12 Jan. 2017 (Fig. 5). All the largest events showed similar 1.8–2.7 km focal depths and consistent mechanism solutions, thereby demonstrating reverse faulting of near north-south striking faults with 30–50° dip angles (Table 1, Fig. 5), and were in very good agreement with the regional stress field and existing faults explored by geophysical and borehole data^18^. Spatial distribution of the largest events in the Y7 pad also agrees fairly well with ΔCFS pattern estimated for favorably oriented reverse faults (Fig. 7). In the Y7 pad, fast diffusion of pore pressure along fault zones is probably required for deeper events, which are partly located in the stress shadow of solid deformation (Fig. 7). However, since the Y7 pad is located outside the range of the portable stations, hypocenter location, especially focal depth, might be poorly constrained.

The productivity of injection-induced seismicity depends on a number of factors including the injection rate, pressure, cumulative injected volume, and the tectonic conditions of the injection site^13, 30^. In order to compare the site-specific productivity of injection-induced seismicity at different sites, we estimated the seismogenic index, defined Σ = log_10_ *N* ≥ _*M*_(t) − log_10_ *V*(t) + *bM*, where *V* is the injected fluid volume and *b* is the seismic *b*-value of the Gutenberg-Richter relation between magnitude and earthquake number^30^. The obtained values are −1.11 for N4 (Fig. 4) and from −1.59 to −1.66 for Y7 (Fig. 5), and are similar to those estimated for injection-induced earthquakes in western Canada^13^.

## Discussion and Conclusions

To summarize, multiple lines of evidence indicate that the hydraulic fracturing operations that began systematically in Dec. 2014 in the Shangluo shale gas site (mainly in the Ning-201 and Ys-108 blocks) caused a rapid increase in seismicity in the region. According to the empirical relationship between moment magnitude and rupture dimension for small and intermediate (0 < *M*_*W*_ < ~5): *M*_*W*_ = log_10_ *A* − 2.0 ^31^ (*A* is rupture area in units of meters squared) events, fractures of a size from approximately 100 to 3000 m are required to produce earthquakes of magnitudes ranging from 2 to 5. Another relationship, *M*_*W*_ = 1.89log_10_ *A* − 5.88, was found to work for microseismicity (*M*_*W*_ < 0) caused by hydraulic fracture opening in shale formations^32^. The shale formation thickness in the Shangluo site is approximately 40−60 m^33, 34^. In fact, microseismic events caused by hydraulic fracture opening have a magnitude less than −1 in the Sichuan Basin^27–29^. Relocated hypocenters show that numerous events (*M*_*L*_ ≥ 1) were located in the overly and underlay formations (Fig. 3). Except for the latest *M*_*W*_4.6 event, mechanism solutions of the earlier 12 largest events (*M*_*W*_ > 3.5) indicate that the largest earthquakes show strike-slip or reverse fault movement along mapped and/or unmapped faults that are favorably oriented with respect to the present-day ambient stress field. Thus, we can convincingly conclude that most *M*_*L*_ ≥ 1 earthquakes discussed in our study occur in response to the reactivation of pre-existing faults. At the same time, newly created fractures within the shale formation might also be potential sources of some small earthquakes.

Together with the earlier results^3, 20, 35^, our studies on injection-induced seismicity in the Sichuan Basin indicate that injection-induced earthquakes (not including microseismicity from hydraulic fracture openings) demonstrate no significant differences from normal tectonic earthquakes in aspects such as mechanism solution, source process, and seismic *b*-value. However, injection-induced seismicity does show some statistical features that are distinguishable from typical tectonic seismicity. The most important one is that injection-induced seismicity shows a very low level of aftershock productivity. This conclusion arises from the ETAS analysis, which showed only 13% Omori-type aftershocks. Thus, it is very important to involve injection details in hazard models.

In the Sichuan Basin, “long-term” wastewater injections (continuing for a few years to several tens of years) into depleted gas reservoirs have caused high levels of injection-induced seismicity resulting in sizable earthquakes up to *M*_*W*_ 5^3, 20^. The present study shows that “short-term” injections (continuing over several months) for shale gas hydraulic fracturing are also very likely to induce *M*_*W*_ 4–5 class earthquakes in sites with similar geological and tectonic conditions within the southern Sichuan Basin.

Many areas where hydraulic fracturing occurs tend to experience predominantly *M*_*W*_ < 2 events, but there are a few places, such as the U.S., U.K., and Canada, that have experienced well-documented earthquakes induced by hydraulic fracturing, with *M*_*W*_ ranging from 2.0 to 4.6^13, 14, 36–38^. Comparing with these cases raises a question as to why earthquake magnitudes associated with hydraulic fracturing operations are so high in the Sichuan Basin. Laboratory AE (Acoustic Emission) studies using rock samples collected from the Sichuan Basin show that major Pre-Triassic sedimentary rocks, including dolomite, Sinian shale, and limestone (Fig. 3), are very hard (high Young’s modulus and fracturing strength) and demonstrate brittle fracturing behaviors^39–41^. Such properties are necessary conditions for maintaining the high-level reservoir stress that leads to seismic fracturing. It is important to note that these pre-Triassic rocks are stronger (can withstand higher differential stresses) than the Silurian mudstone/shale formation.

Our numerical analysis shows that the significant ΔCFS resulting from a typical hydraulic fracturing treatment is limited to an area within 2–3 km from the fracturing well. Pre-existing faults with lengths of up to a few kilometers in that area that also possess significant ΔCFS levels are a necessary precondition for moderate-sized fracture-induced earthquakes (*M*_*W*_ 4–5). Small and immature faults exist widely in the brittle formations in the southern fold zones of the Sichuan Basin^35, 39^. Geophysical and borehole data show that the Shangluo shale gas site, which is located in the fold zone, has a relatively high density of blind faults^18^. As seeing from the cross sections in Fig. 3 the earthquakes continue both downward into the crystalline basement and upward well above the injection interval. At pad Y7, it appears from the event locations on the cross section that fluid pressure entered a major fault that cuts through the pre-Triassic rocks and down into the crystalline basement. Thus, we suggest that (1) strong and brittle Pre-Triassic sedimentary rocks; (2) critical regional stress; (3) widely existing faults; (4) there are insufficient top and bottom seals and/or no fracturing barrier between the shale formation and the rocks above and below are the geologic reasons why earthquake magnitudes associated with hydraulic fracturing operations are so high in the study area. In some wells, the production practices are inadequate to keep the hydrofracs within the target reservoir formation. It is very interesting that the largest earthquakes (M4+) are happening within the pre-Triassic sedimentary rocks, not in the basement (Fig. 3). Depth of the basement (greater than 6 km in the study area) and widely distributed detachments in the sedimentary formations may have a role, and further study is required.

It is worth noting that the Weiyuan shale site, located in the central uplift of the Sichuan Basin (Fig. 1-B), also shows a high level of injection-induced seismicity. However, despite the fact that hydraulic fracturing treatments in the same Longmaxi shale formation were performed with higher fluid pressure (the shale formation in the Weiyuan site has greater depth) than used at the Shangluo site, the largest earthquake observed so far was a *M*_*W*_ 3.4 event (Supplementary Fig. 22). When compared with the southern fold zone, sediment deformation in the central uplift of the Sichuan Basin is relatively weak and fewer faults in the sedimentary layers, so a lower probability of *M*_*W*_ 4 class earthquakes is expected, which is in agreement with our suggestions.

The present analysis, especially the numerical model for THM coupled simulation, could be significantly improved by including additional data, such as micro-seismic, 3D geophysical, and detailed injection data. A detailed distribution of pre-existing faults will be the most important factor for creating a subsequent hazard model. Shale gas development is on the rise in the Sichuan Basin including the Shangluo site and its surrounding areas. In the N201, Ys108, and Ys111 blocks, surrounding the Shangluo town, more than 200 hydraulic fracturing wells will open for operation in the coming years^15^. In addition, the N201 and Ys108 shale gas blocks have three injection wells for injecting wastewater (coproduced water with gas production and hydraulic fracturing flowback water) into the Maokou and Qixia limestone formations, overlying the Longmaxi shale formation (Fig. 3)^15^. These wells will probably open for use in the near future. Thus, we assume that the study area will encounter with increasing likelihood additional injection-induced seismicity.

Taking these points into consideration, it would be beneficial for academic, oil industry, and regulator communities to work collectively in order to elucidate the governing factors behind the high level of injection-induced seismicity in the southern Sichuan Basin, thereby allowing shale gas hydraulic fracturing to be conducted effectively and safely. It will also be a challenge to develop a hazard model that anticipates where fault reactivation and earthquakes may be induced in response to changing industrial drivers^8^. Our results are expected to be helpful for conducting risk assessments at other sites with similar geological and tectonic conditions.

## Methods

### Epidemic-Type Aftershock Sequence Model

To examine the statistical features of the seismicity, we applied the epidemic-type aftershock sequence (ETAS) model, which is useful for extracting a fluid signal from seismicity data^3, 20–22^. In the ETAS model, the total occurrence rate is described as the sum of the forcing (background) rate *λ*_0_(*t*) and the Omori’s law aftershocks $\nu (t)$ triggered by all preceding earthquakes:

1 $$\begin{array}{c}\lambda (t)={\lambda }_{0}(t)+\nu (t)\\ \nu (t)=\sum _{\{i:{t}_{i} < t\}}{K}_{0}{e}^{\alpha ({M}_{i}-{M}_{c})}{(t-{t}_{i}+c)}^{-p}\end{array}$$

where *M*_*c*_ is the estimated cut-off magnitude of completeness, *α* is a constant that specifies the degree of magnitude dependence, and *p* and *c* are constants of Omori’s law. For injection-induced seismicity, the forcing rate depends on injection factors, and thus a time-varying forcing rate should be used^20, 42^. Model parameters were estimated by minimizing the Akaike information criterion (AIC)^20^. We carried out the robust estimation of the standard errors of the ETAS parameters using Monte Carlo simulations. Following the procedure set forth in a previous study^43^, 1,000 ETAS model simulations were run, and the ETAS parameters of each simulated earthquake sequence were estimated. For each run, ETAS parameter estimates of the simulated earthquake sequence were obtained. The standard error could then be estimated from these simulations by using the root mean square of the errors of the estimated parameters from the simulations as

2 $$SE=\sqrt{{{\sum }_{i=1}^{N}({\theta }_{i}-{\theta }_{true})}^{2}/N},\quad \theta =\{{\lambda }_{0}(t),K,c,p\},$$

where *θ*_*i*_ and *θ*_*true*_ are the estimated parameters from the *i* ^th^ simulation and the real data, respectively.

### Moment Tensor Inversion

Earthquakes of a magnitude greater than 3.5 in the study area could be recorded well by numerous broadband seismic stations at distances of up to 300–500 km. Thus, in order to obtain reliable focal depth and mechanism solutions for the largest earthquakes, we inverted the mechanism solution and the moment tensor using the generalized cut and paste (gCAP) method, in which the full waveforms of body and surface waves recorded by broadband seismometers are used^23, 44^. It has been shown that ‘the method of using amplitude spectra of surface waves’ can determine the focal depth of an earthquake at an accuracy level of a few kilometers^45^. Particularly, surface waves impose a robust constraint on focal depth estimations for shallow earthquakes (Supplementary Fig. 7). The full moment tensors are estimated by a grid search with respect to the moment magnitude (step of 0.01) and the strike, dip, and rake angles (step of 5°) of the faults and slip orientations. The general seismic potency tensor is decomposed into double-couple (DC), isotropic (ISO), and compensated linear vector dipole (CLVD) components^23^.

The original seismograms were typically filtered with corner frequencies (0.02, 0.15) or (0.02, 0.1). Unit weight was applied for all phases. The results of seismic ambient noise tomography^46^ were used to construct a mean 1D velocity model (M-2, preferred model) for gCAP inversion (Supplementary Fig. 6). A reference model (M-3) was also tested for assessing the uncertainties of moment tensor inversion (Supplementary Fig. 6). Since the results of the two velocity models are quite similar in both mechanism and focal depth (in a narrow range of 1.8–4.0 km) (Supplementary Table 1), thereby indicating that uncertainty due to velocity error is insignificant, we focused on the preferred M-2 model results in this study. In total, we obtained reliable inversions for all 13 earthquakes having moment magnitudes greater than 3.5 (Table 1).

### Hypocenter Relocation

We used phase data manually picked and compiled by the YEB method and Hypocenter Double-Difference (HypoDD) program^24^ to relocate the earthquake hypocenters. A mean velocity model (M-1) of the study area and was used (Supplementary Fig. 6). In total, we utilized up to 20 stations, 4,711 selected events, 224,442 differential P times, and 222,890 differential S times.

### Seismogenic Index

The productivity of injection-induced seismicity depends on several factors, including injection rate, pressure, cumulative volume injected, and regional and local tectonic conditions of an injection site. The seismogenic index, defined as

3 $${\rm{\Sigma }}={\mathrm{log}}_{10}{N}_{\ge M}({\rm{t}})-{\mathrm{log}}_{10}V({\rm{t}})+bM$$

where *V* is injected volume of fluid and *b* is the seismic *b*-value of the Gutenberg-Richter relation between magnitude and earthquake number, provides a measure of site-specific seismic productivity^30^.

We were able to estimate the seismogenic index of two sites, N4 and Y7. We visited well pad N4 on 28 Feb. 2016 when hydraulic-fracturing was in progress. Four white boards with detailed information about the wellbores and injections were available for public viewing at the pad gate. N4 has three southward and three northward horizontal wellbores, in which zipper fracture stimulations were performed between Feb. and June 2016. Each of these wellbores extend horizontally 1.5 to 2 km, and each contains more than 22 (up to 27) stages. The largest earthquake in N4, which had a magnitude of *M*_*L*_ 3.1, occurred 40 days after hydraulic fracturing began. The total volume of fluid injected into the six wellbores during operations was approximately 252,000 m^3^. Average injection rate was approximately 10–14 m^3^/min, and the estimated seismogenic index was −1.11.

Injections in the Y6, Y8, and Y7 sites are responsible for the earthquakes surrounding the Y7 pad. Treatments at Y7 and Y6 are ongoing. The total volume of fluid injected during the period from Sept. 2016 to Feb. 2017 was approximately 120,000 m^3^. The estimated seismogenic indexes are −1.59 for the Y8 stimulation and −1.66 for all data collected thus far.

### Coulomb Failure Stress

In order to estimate stress redistribution patterns resulting from hydraulic fracturing injections, we carried out coupled THM analysis using two simulators: TOUGH2 and FLAC3D. TOUGH2 is a multiphase reservoir simulation program developed by the Lawrence Berkeley National Laboratory (LBNL) in the U.S. FLAC-3D is a commercial software package for stress analysis that is based on the standard poroelastic equations explicitly using finite difference method^47^. With couplers^48^, the “TOUGH-FLAC” approach has been used in the analysis of fluid flow accompanied deformation in geothermal studies, and in natural fluid pressure-triggered seismicity^35, 49–51^.

Based on the geological profile shown in Fig. 3 of the main text, we built a simplified model containing four layers (Fig. 6) of different mechanical and hydraulic properties (Supplementary Table 2), which were determined through a number of runs, to match the observed data (the maximum injection pressure and surface uplift). The X-axis of the model’s coordinate system is parallel to the maximum horizontal stress (azimuth of 110°), and thus, the Y-axis is parallel with the horizontal wellbores.

The far field boundaries are placed at a distance of 10 km in order to approximate infinite boundaries. The in-site stress values are installed in all zones, and also applied as loads acting on the far-field boundaries. Since we focused on differential stresses due to injections, precise in-site stress measurement was not required. The vertical stress *σ*_*V*_ was calculated from assumed density values. Based on fault slip-tendency analysis, the maximum and minimum horizontal stress values were assumed as *σ*_*H*_ = 2.32 *σ*_*V*_, and *σ*_*h*_ = 1.1 *σ*_*V*_, respectively. Thus, the most favorably oriented faults were critically stressed. Such a stress regime allows both strike-slip and thrust faulting according to the strike and dip of a fault. Thus, the assumed stress regime is consistent with the fact that both strike-slip and reverse mechanisms were obtained in the study area. Our model has dimensions of 20, 20, and 6 km in the X, Y, and Z (depth) directions, respectively, and is divided into grids by varying steps from 20 to 2,000 m according to distance increases from the center point. The bottom boundary conditions are rollers, the four side boundaries are fixed, and the top boundaries are free. Initially hydrostatic pore pressure was assumed. For short-term injection, results are insensitive to boundary flow conditions.

We began by simulating change of the Coulomb failure stress due to hydraulic fracturing in a single well and single stage in order to examine ΔCFS patterns on favorably oriented strike-slip and reverse faults, which were the source faults of the largest earthquakes observed in the study area. In total, 1,800 m^3^ of water was injected at a rate of 10 m^3^/minute from the “well” zone located at the center (x = 0, y = 0) and vertically in the middle of the shale formation. Numerical results show a maximum injection pressure of approximately 65 MPa and surface deformation up to a few millimeters, which was consistent with the observed data^17^.

We then simulated ΔCFS evolution following multistage zipper fracturing in two wells. As can be seen from estimated ΔCFS distributions on favorably oriented strike-slip and thrust faults after 10-stage hydraulic fracturing, it is important to note that 1) there were different stress patterns for strike-slip and thrust faults, and that 2) significant ΔCFS (amplitude greater than 0.1 MPa) may extend for a number of kilometers due to solid deformations (Fig. 7).

## Electronic supplementary material

Supplementary Information 1-9
