# Supplementary material for: Fault reactivation and earthquakes with magnitudes of up to Mw4.7 induced by shale-gas hydraulic fracturing in Sichuan Basin, China
Source: Sci Rep. 2017 Aug 11;7:7971. doi: 10.1038/s41598-017-08557-y (PMC5554178; doi:10.1038/s41598-017-08557-y)
Supplement: Supplementary file 1 — Supplementary Information 1-9 [file 41598_2017_8557_MOESM1_ESM.pdf]

Fault reactivation and earthquakes with magnitudes of up to M_W 4.7 induced by shale-gas hydraulic fracturing in Sichuan Basin, China

Xinglin Lei[1*], Dongjian Huang[2], Jinrong Su[3], Guomao Jiang[2], Xiaolong Wang[4], Hui Wang[2], Xin Guo[4], Hong Fu[5]

[1] Geological Survey of Japan, AIST, Tsukuba 305-8567, Japan
[2] Yibin Earthquake Mitigation Administration, Yibin 64400, China
[3] Earthquake Monitoring Centre, Sichuan Earthquake Administration, Chengdu 610041, China
[4] Department of Science and Technology, Chongqing Earthquake Administration, Chongqing 401137, China
[5] Center for Research and Prediction, Yunnan Earthquake Administration, Kunming 650224, China
*Corresponding author, Phone: +81-29-8612468 E-mail: xinglin-lei@aist.go.jp

Supplementary Information 1:

An overview of Injection Induced Seismicity in Sichuan Basin

The Sichuan Basin, which is located in the northwest portion of the South China block, is a relatively stable continental region. Major earthquakes in the Sichuan Basin primarily occur within the Zigong and Yibin areas, where pumping and water injection have been routinely performed for centuries in order to dissolve rock salt from deep wells. The Sichuan Basin is also a major national source of petroleum. Since the 1980s, progressively increasing amounts of the wastewater that is coproduced with natural gas have been injected into deep wells in some of the region's depleted gas reservoirs. During the same time period, a number of earthquake clusters have been observed, with sizable earthquakes ranging up to M_W 4-5 ('C' and 'D' in Fig. 1). The timings, locations, and occurrence patterns of these earthquakes in statistical models convincingly suggest that they were injection-induced by the wastewater disposal[1,2].

The Sichuan Basin is also one of the areas with the greatest potential for shale gas development[3-6], and the widely distributed Palaeozoic and Proterozoic shale formations in the basin are attractive targets for shale gas reservoir developers. The shale gas industry breakout in China began in 2010, initiated by the build-up of the first Silurian shale gas well in the Weiyuan area, southwest of the Sichuan Basin (`B` in Fig. 1). Systematic shale gas hydraulic fracturing in horizontal wells in the Shangluo shale gas site ('A' in Sup. Fig. 1, study area of this paper) began in 2014.

North of the study area, there is a deep well salt site ('D' in Sup. Fig. 1) into which water has been repeatedly injected since 2002 for the purpose of dissolving and producing salt. Such injections are also suspected of having induced significant earthquakes, including several $M_W > 4.0$ events. However, to date, the two earthquake clusters ('A' and 'D' in Sup. Fig. 1) remain spatially separated, so injections at the salt site do not appear to have any effect on our study area. In the

west of the study area ('E' in Sup. Fig. 1), an increased number of events were observed in 2002 and 2003, and those events are suspected of being related to coal mining activities.

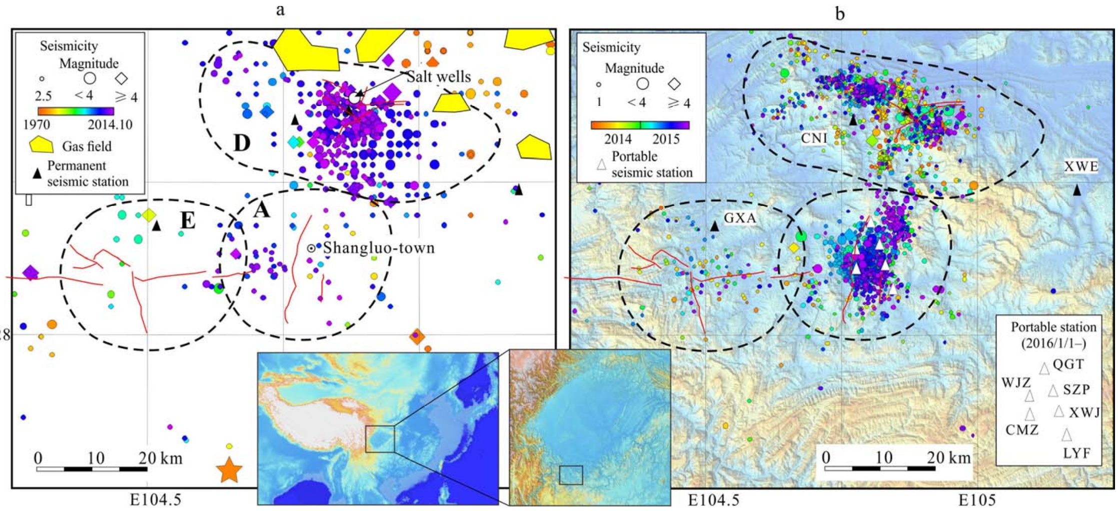

Supplementary Figure 1: Earthquakes observed during periods since 1970 to Oct. 2014 (a) and from 2014 to 2016 (b) at the Shangluo shale gas site ('A') and surrounding regions. The dense cluster centered at the Changning well salt site ('D') is related to deep well injections for dissolving and producing salt[7]. The 'E' mask covers a region of coal mines, in which mining-induced seismicity was observed. The map insets highlight portable stations, which were installed and began operating on 1 Jan. 2016. This map was created using the free software GeoTaos_map (developed by Xinglin Lei; https://staff.aist.go.jp/xinglin-lei/) and finished with the software CorelDRAW X8. (Copyright (c) 2016 Corel Corporation. All rights reserved.)

Supplementary Information 2:

Seismic Stations, Earthquake Catalogue, and Temporary Observation

The Sichuan seismic network has four nearby stations located within 20 to 40 km from the study area (Sup. Fig. 2). The estimated magnitude of completeness is 2.0. Noting the abnormal seismicity and its possible link with the hydraulic fracturing activities, the Yibin Earthquake Mitigation Administration (YEA) had installed six portable seismic stations by the end of 2015 (Sup. Fig. 3). Since then, the magnitude of completeness has decreased to 1.0.

In the present study, two datasets were used. One is the China Earthquake Data Centre (CEDC) catalogue, which was used for ascertaining background seismicity in the study area and surroundings. This catalogue is publicly available from data.earthquake.cn. The other dataset is the phase data, which were manually extracted and compiled by YEA from seismograms observed by nearby and portable stations, and which has been used for detailed seismicity studies since 2014.

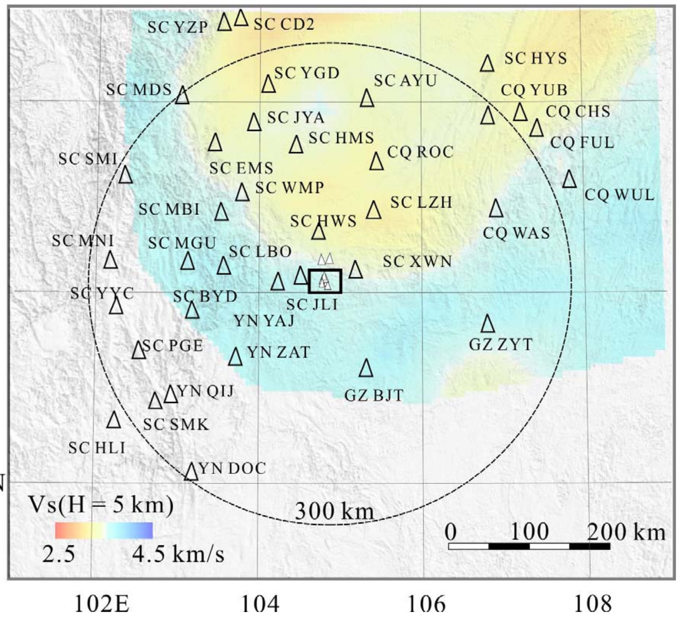

Supplementary Figure 2: Distribution of seismic stations and S velocity. Large triangles indicate broadband stations used for mechanism solutions. Small triangles indicate nearby short-period stations. The background colors show S-wave velocities at a depth of 5 km, estimated from seismic ambient noise tomography[8]. The rectangle in the center indicates the study area. This map was created using the free software GeoTaos_map (developed by Xinglin Lei; https://staff.aist.go.jp/xinglin-lei/) and finished with the software CorelDRAW X8. (Copyright (c) 2016 Corel Corporation. All rights reserved.)

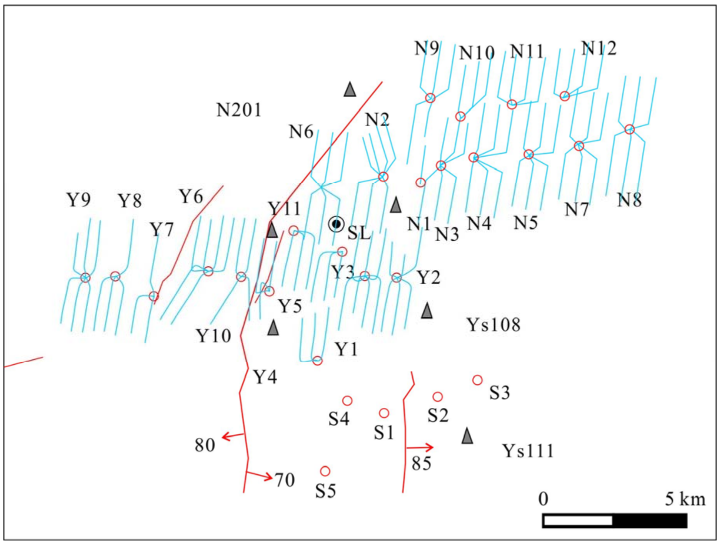

Supplementary Figure 3: Locations of well pads (red circles) and portable seismic stations (triangles) in Shangluo-town (SL) and the surrounding environs. N201, Ys108, and Ys111 indicate shale gas blocks that were named as the evaluation wells. N#, Ys#, and S# indicate horizontal well pads in N201, Ys108, and Ys111, respectively. The blue lines show horizontal well traces, while the red lines show mapped active faults compiled by the YEA.

Supplementary Information 3: Field Surveys and Injection Data

In the Shangluo-town shale gas site, hydraulic fracturing treatments have been carried out in two blocks, N201 and Ys108 (Sup. Fig. 3). These blocks have now been recognized as the first vertical well sites drilled for evaluation purposes. Basic information regarding these shale gas construction projects, including environmental aspects, geological conditions, well data, injection schedules, average injection pressure and injection rate, and wastewater treatment, are publicly available[9]. Except for some published cases, unfortunately, detailed injection data are not available to the public. Nevertheless, we managed to gather significant amounts of hydraulic fracturing information through frequent field surveys. The location of major integrated operating platforms (well pads) and traces the horizontal wellbores of each pad (shown in Sup. Fig. 3) were digitized from a public report[9] and verified through field investigation. At each well pad, several whiteboards have been set up to provide the public with basic information about the pad, including vertical depth, the horizontal extension of all wellbores, and the drilling and hydraulic fracturing schedules. As can be seen in Sup. Fig. 4, fluctuations in earthquake occurrences closely reflect major hydraulic fracturing periods.

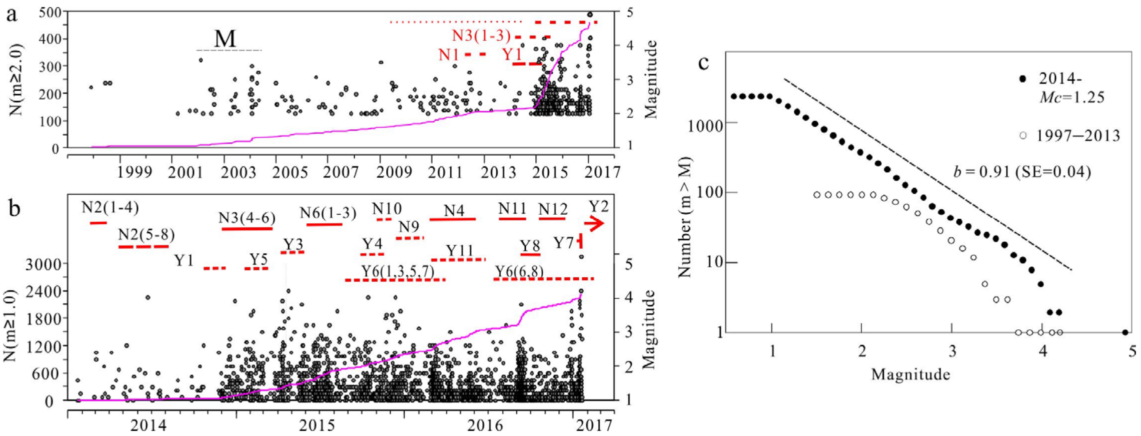

Supplementary Figure 4: Earthquakes and major hydraulic fracturing periods in the study area, i.e., Region A of Sup. Fig. 1. (a) Earthquakes and major hydraulic fracturing time windows at well pads in the N201 and Ys108 shale gas blocks in Shangluo town ('A' in Sup. Fig. 1). (b) Detailed and later time portion of (a). N# and Ys# indicate horizontal well pads in N201 and Ys108, respectively (see Sup. Fig. 4 for well locations). Red dotted lines indicate hydraulic fracturing for extraction in vertical or horizontal wells. Red and red dashed lines indicate systematic hydraulic fracturing in horizontal wells. Dotted and dashed lines indicated the corresponding time windows are rough estimations from field surveys. (c) Magnitude–frequency distributions for different periods. Since 2009, earthquakes have shown clear responses to hydraulic fracturing operations.

Supplementary Information 3: Monte Carlo simulation of ETAS

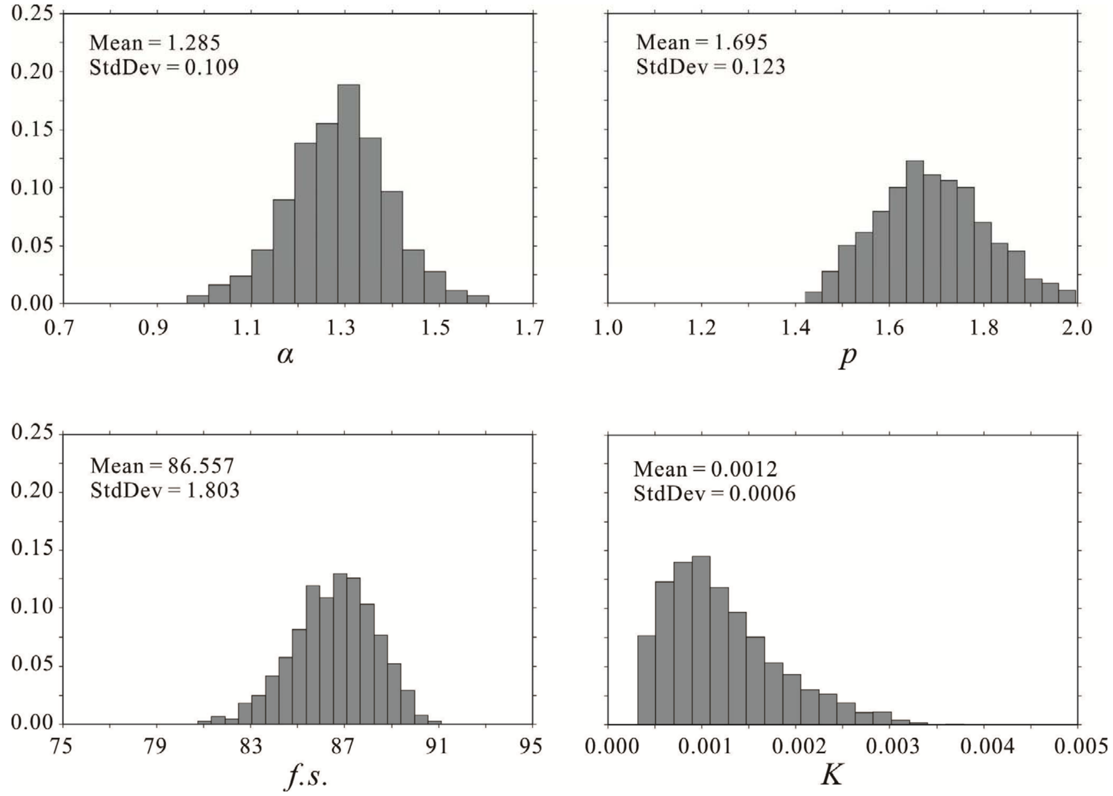

Supplementary Figure 5: Results of Monte Carlo simulations for estimating the standard errors of the ETAS parameters (*f.s.*, *K*, *α*, *p*) shown in Fig. 2c. In total, 1,000 ETAS model simulations were run, and the ETAS parameters of each simulated earthquake sequence were estimated.

Supplementary Information 4: Local and Regional Velocity Models

In order to relocate seismic events and inverse full moment tensor of the largest events, we constructed two velocity models based on the results of seismic ambient noise tomography[8]. The first model (M-1) is based on mean S velocities (*Vs*) of the study area and was used to relocate the earthquake hypocenters. The second model (M-2) is based on the mean profile of a wider area, with distances of up to 300 km from the study area (Sup. Fig. 6). M-2 was the preferred model for moment tensor inversion. In both models, the P velocity (*Vp*) is calculated from *Vs* by assuming a *Vp*/*Vs* ratio of 1.73. An additional model, M-3, was also tested to assess moment tensor inversion uncertainties.

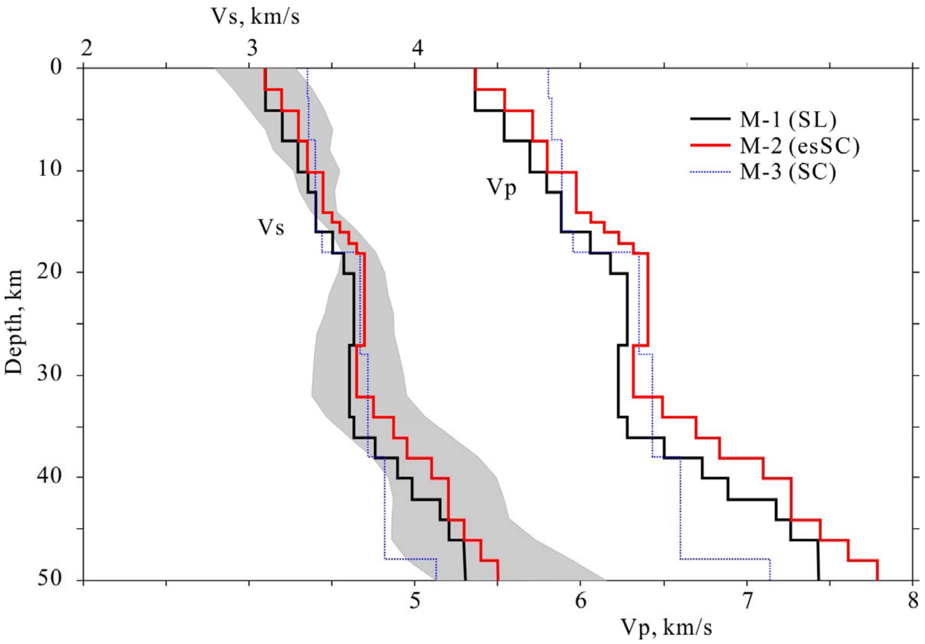

Supplementary Figure 6: Velocity model used for earthquake relocation and moment tensor inversion. The S velocity (*Vs*) is based on results of seismic ambient noise tomography (see text for details). The shaded region shows the minimum and the maximum S velocities within a distance of approximately 300 km from the study area. The P velocity (*Vp*) is calculated from *Vs* by assuming a *Vp*/*Vs* ratio of 1.73. M-1 represents local mean velocities of the study area and was used to relocate the seismic events. M-2 is the preferred model, which was estimated from the mean profile of the wide area, for moment tensor inversion. M-3 is a reference model used to examine the uncertainty of moment tensor inversion.

Supplementary Information 5: Moment Tensor Inversion

Supplementary Figure 7 shows synthetic seismograms calculated for the focal depth of a virtual event. These indicate that surface waves impose a robust constraint on focal depth estimations for shallow earthquakes. In total, we obtained reliable inversions for all 13 earthquakes having moment magnitudes greater than 3.5. The results obtained using the preferred and reference models are listed in Sup. Table 1. Since the results of the two velocity models were quite similar in both mechanism and focal depth (in a narrow range of 1.8-4.0 km), thereby indicating that uncertainty due to velocity error is insignificant, we focused on the preferred M-2 model results in this study (Table 1 of the main text).

Sup. Figs S7-S14 show examples of misfit error against assumed focal depth and waveform match for four earthquakes with different mechanisms: 1) strike-slip with significant reverse component; 2) strike-slip dominated; 3) reverse dominated; and 4) reverse with a strike-slip component. Sup. Fig. 8 shows a comparison of misfit error against the assumed focal depth using different velocity models for the 14 Oct. 2015 M_L 4.0 earthquake. Sup. Fig. 9 shows a comparison of observed and synthetic velocity waveforms calculated for the best-fit focal depth. As can be seen

in this figure, most observed waveforms are well represented by the estimated source parameters. The estimated moment magnitude was M_W 3.7 and two nodal planes of the best-fit focal depth (2.74 ± 0.03 km) were estimated as (strike, dip, rake) = (339, 65, 29) / (236, 64, 152), thereby indicating a strike-slip offset with a significant reverse component.

Sup. Figs 10 and 11 show the results of the M_L 4.2 event that occurred on 25 Apr. 2015. The estimated moment magnitude was M_W 4.05 and two nodal planes of the best-fit focal depth (2.96 ± 0.02 km) were estimated as (strike, dip, rake) = (144, 76, 7) / (52, 83, 166), thereby indicating that it was a typical strike-slip dominated event.

Sup. Figs 12 and 13 show the results of the M_L 3.8 event that occurred on 10 Sept. 2016, which demonstrated a pure reverse faulting mechanism with a focal depth of 2.7 km. Sup. Figs 14 and 15 show the results of the largest M_L 4.9 event, which occurred on 28 Jan. 2019, and which demonstrated a reverse faulting mechanism with a strike-slip component. The estimated focal depth is 1.8 km. Sup. Figs 16 and 17 show the results of the latest M_L 4.9 event, which occurred on 4 May 2017, and which demonstrated a strike slip faulting mechanism and focal depth of 2.4 km.

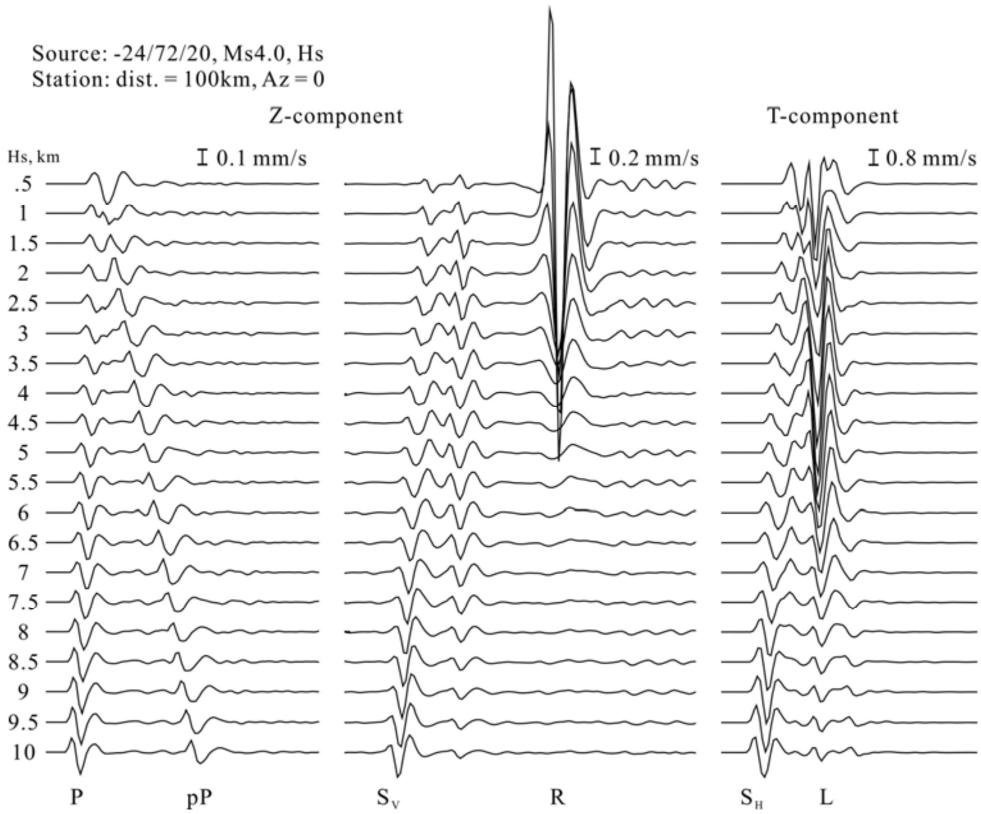

Supplementary Figure 7: Synthetic seismograms calculated for a virtual M_w 4.0 source fault defined by (strike, dip, rake) = (-24, 72, 20) and focal depth (Hs) from 0.5 to 10 km. The preferred M-2 velocity model (Sup. Fig. 6) is used. The station is located at a distance of 100 km and an azimuth of 0° from the source.

#	EQ	FM1	FM2	G_{iso}, G_{vlcd}	M_W	M_L	*H*, km	*H**, km
1	2015/04/11	339/63/26	237/67/150	-0.04, -0.00	3.54	3.9	3.3	2.6±2.5
		154/65/9	60/82/155	-0.00, -0.00	3.60		4.1	
2	2015/04/25	144/76/71	52/83/166	+0.02, +0.00	4.05	4.2	3.0	5.1±0.9
		44/78/6	53/84/168	-0.02, -0.01	4.10		4.8	
3	2015/04/30	145/76/9	53/81/166	-0.00, -0.01	3.51	3.7	2.9	2.7±0.8
		145/80/10	53/80/170	-0.01, -0.01	3.60		3.3	
4	2015/05/23	-22/64/27	235/66/151	-0.12, -0.01	3.52	3.8	4.0	5.4±0.8
		335/67/22	236/70/15	-0.14, -0.01	3.60		4.4	
5	2015/10/14	339/65/29	236/64/152	+0.04, +0.00	3.72	4.0	2.7	4.2±1.2
		-24/72/20	240/71/161	-0.02, -0.01	3.80		3.3	
6	2015/12/12	149/70/7	57/83/160	+0.07, +0.00	3.59	4.0	2.5	3.8±0.6
		-29/72/17	236/74/161	-0.03, -0.03	3.70		4.8	
7	2016/09/10	-14/60/93	160/30/85	-0.2, -0.03	3.66	3.8	3.4	2.94±0.3
		-27/46/74	175/46/106	-0.01, -0.00	3.70		2.5	
8	2017/01/15	15/42/74	216/50/104	0.04, 0.02	4.07	4.3	2.4	3.07±0.5
	18:05:35	20/36/82	210/54/96	-0.01, 0.01	4.09		3.0	
9	2017/01/15	14/64/85	205/26/100	0.00, 0.00	3.86	3.7	2.7	3.70±0.6
	19:20:54	7/52/79	205/39/104	0.00, 0.00	3.84		2.2	
10	2017/01/15	19/61/82	216/30/105	0.00, 0.00	3.84	3.8	2.4	6.7±0.4
	19:29:17	10/49/71	218/44/111	0.00, 0.00	3.82		2.2	
11	2017/01/18	355/49/65	210/47/116	0.00, 0.00	4.11	4.3	1.8	3.3±0.4
	22:35:14	-15/41/49	214/60/120	0.00, 0.00	4.13		1.8	
12	2017/01/20	-6/51/73	200/42/110	0.04, 0.02	4.67	4.9	1.8	5.1±0.1
		-5/36/75	194/55/101	0.03, 0.00	4.67		1.9	
13	2017/05/04	186/84/14	95/76/174	-0.04, -0.04	4.56	4.9	2.4	
		3/77/-9	95/81/-167	0.03, 0.00	4.58		2.5	

Supplementary Table 1: Mechanism solutions for the largest earthquakes with $M_W > 3.5$. Columns FM1 and FM2 show the strike/dip/rake of the two nodal planes. G_{iso}, G_{vlcd} are, respectively, the squared ratios of the scalar potency of the ISO, and the CLVD component to the total scalar potency, representing their relative strengths[10]. H and M_W are central moment depth and moment magnitude of the best solution. For comparison, M_L from the catalog and focal depth (H*) and its standard error determined by the double differential relocation method are also shown. The focal depths are related to a mean surface level.

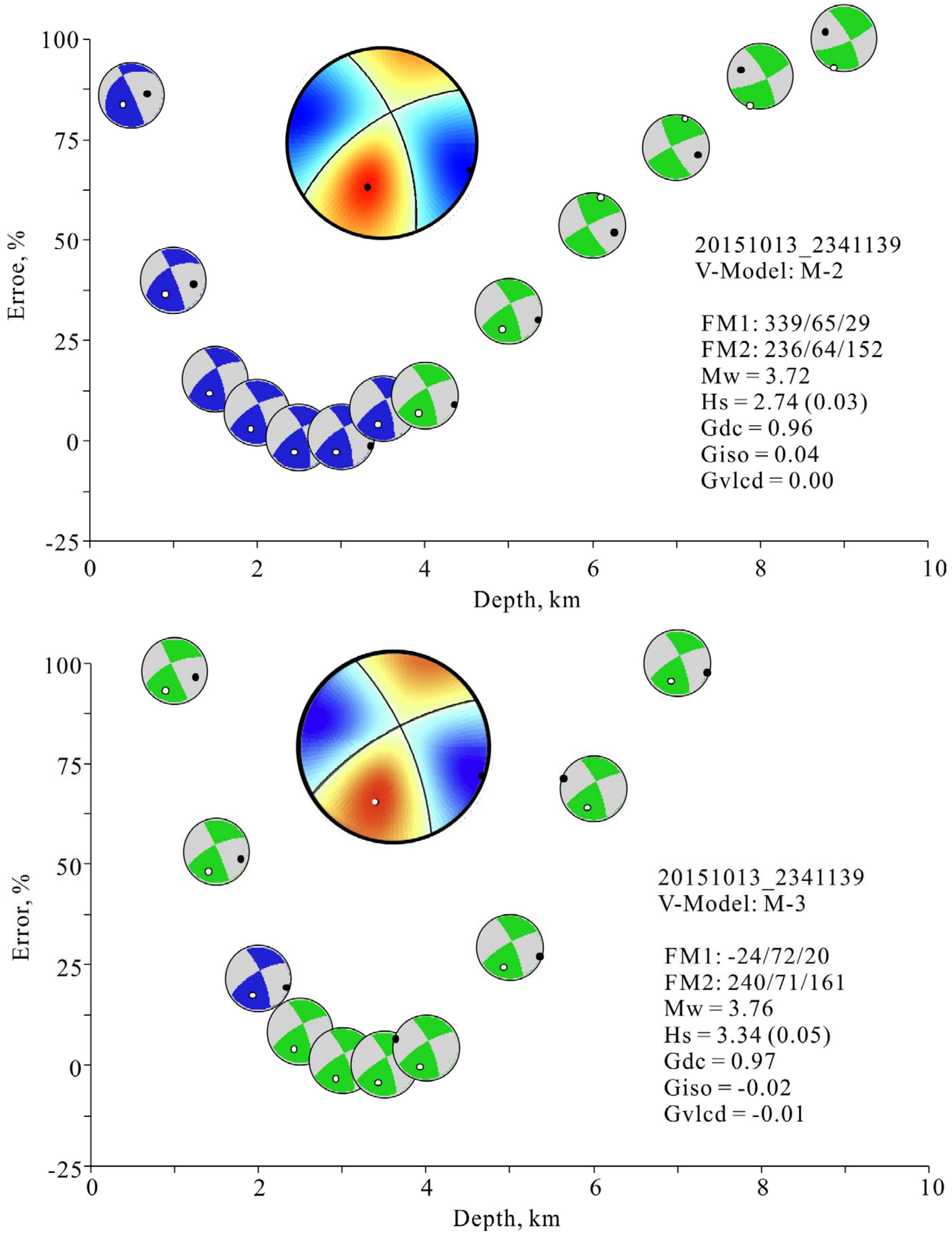

Supplementary Figure 8: Compression of misfit error of moment tensor inversions by the generalized cut and paste (gCAP) method using two velocity models against assumed focal depth for the 14 Oct. 2015 M_L 4.0 earthquake, which yielded strike-slip with a significant reverse component. The green/blue mechanisms indicate a "strike-slip"/"reverse" faulting mechanism. In this example, the strike-slip and reverse components are almost equal. The color scale in the large focal mechanisms indicates the P wave radiation pattern.

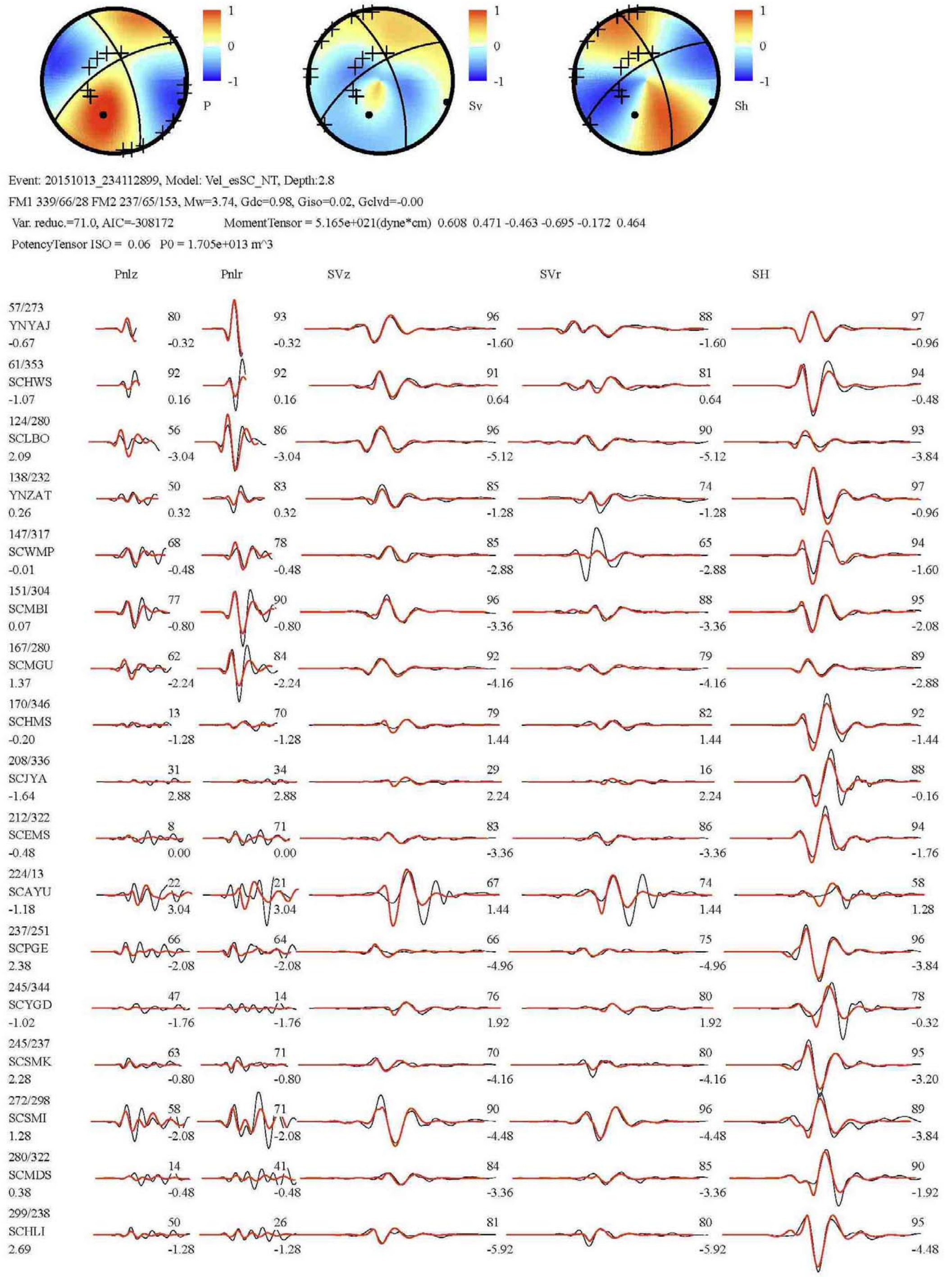

Supplementary Figure 9: Waveform match for the 14 Oct. 2015 M_L 4.0 earthquake. The color bars on the focal mechanisms indicate the normalized values of radiation strength of P/Sv/Sh. The black and red traces indicate the observed and synthetic velocity waveforms, respectively. The numbers above the station code are source-station distance and azimuth. The numbers below the station code are the time shift needed to align data and synthetic approximately (positive shift means synthetic is earlier). The numbers above and below each trace segments are the cross-correlation coefficients (%) and the time shifts (in seconds), respectively.

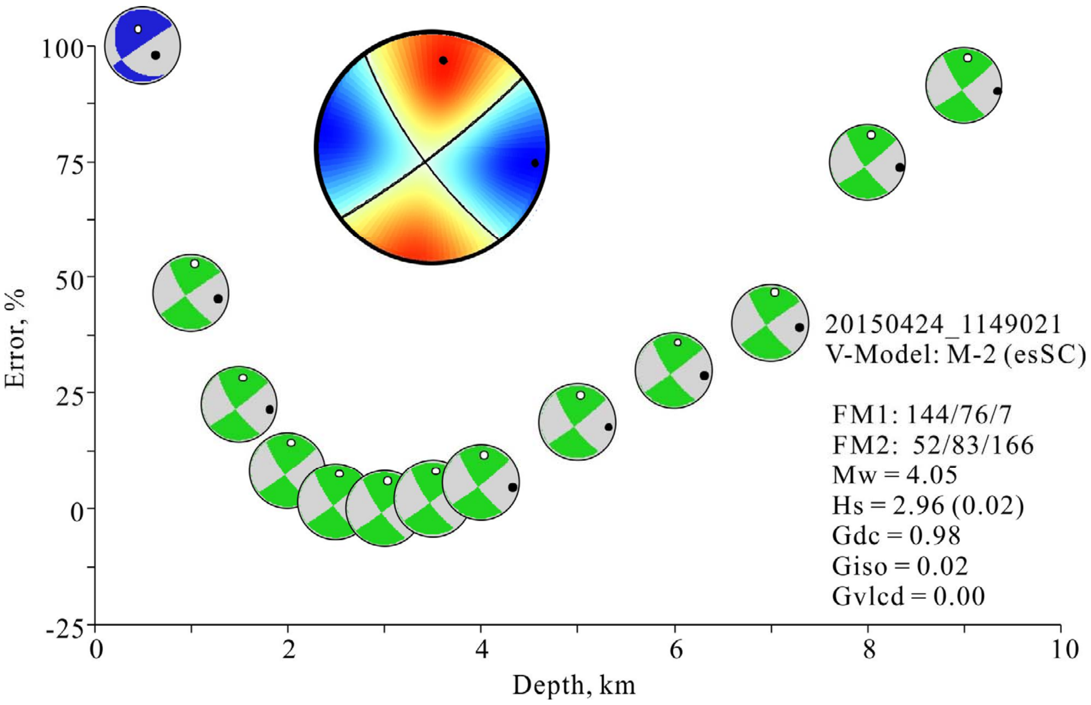

Supplementary Figure 10: Misfit error of moment tensor inversion by gCAP method against assumed focal depth for the 25 Apr. 2015 M_L 4.2 earthquake, which was a typical strike-slip event.

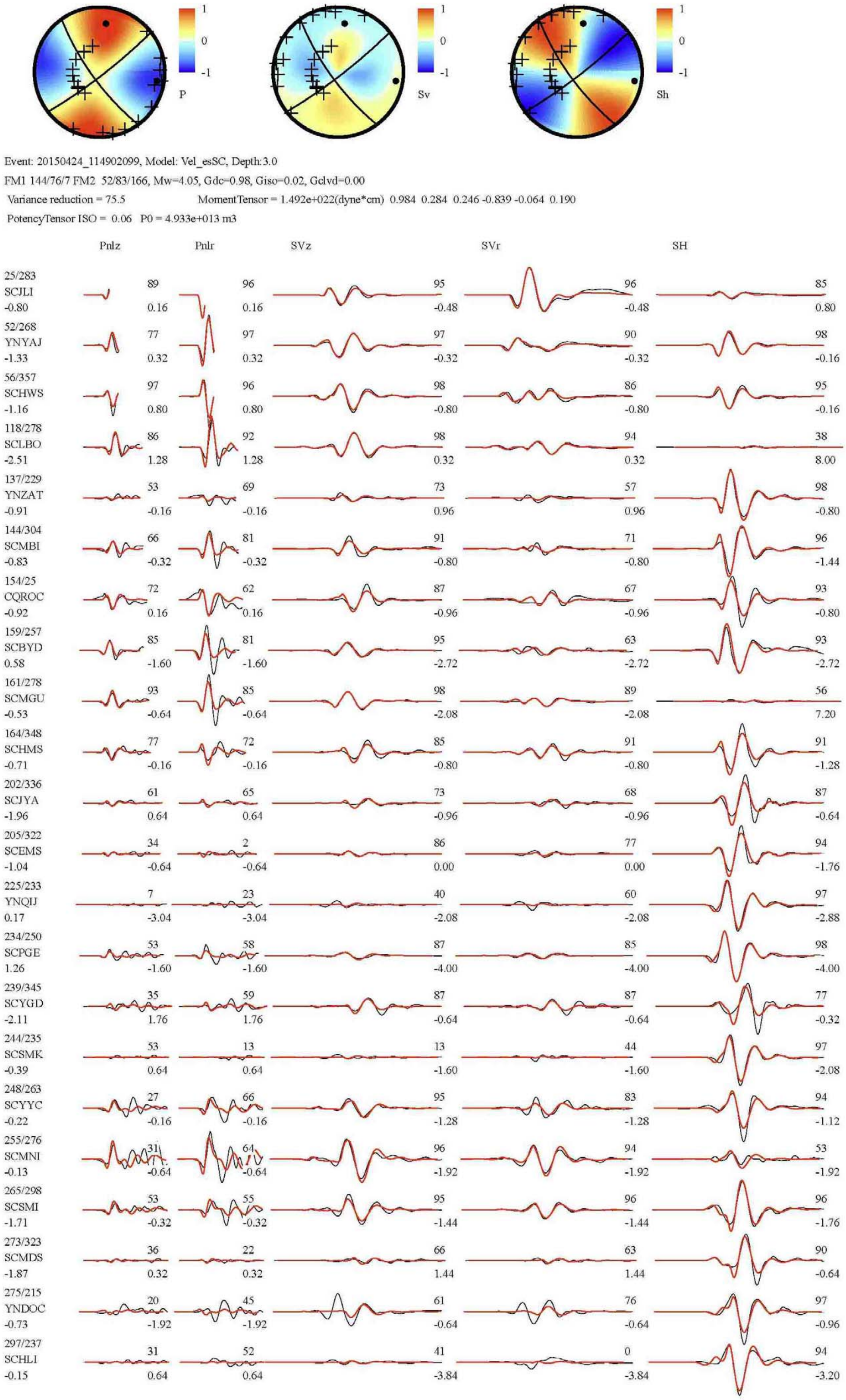

Supplementary Figure 11: Waveform matches for the 25 Apr. 2015 M_L 4.2 earthquake. See Sup. Fig. 9 caption for details.

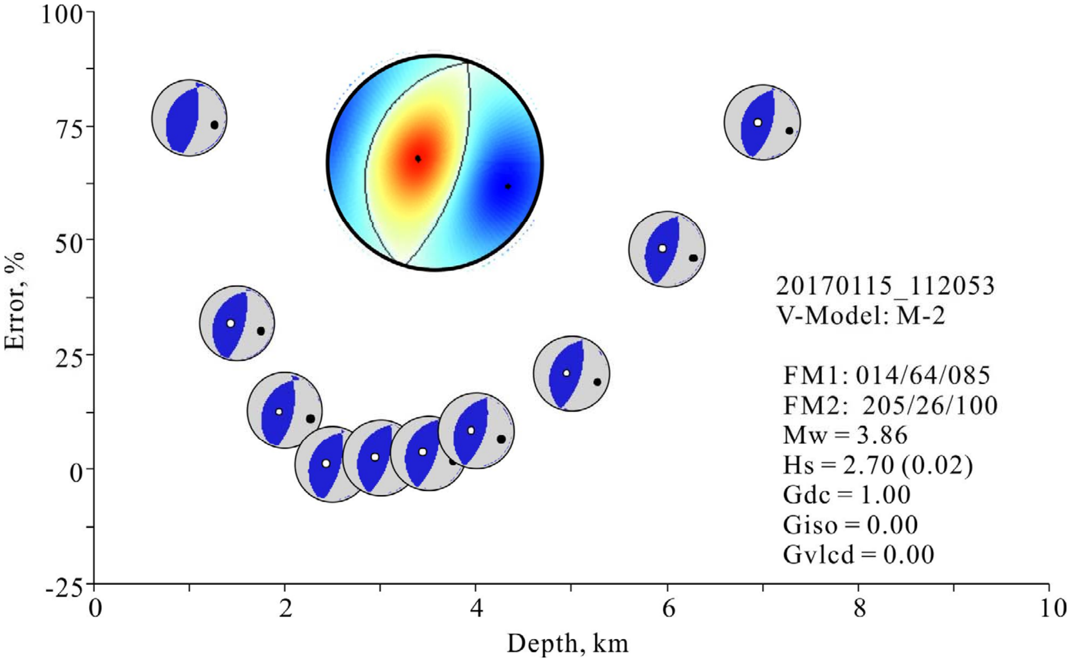

Supplementary Figure 12: Misfit error of moment tensor inversion by gCAP method against the assumed focal depth for the 15 Jan. 2017 M_L 3.7 earthquake, which was a typical reverse event.

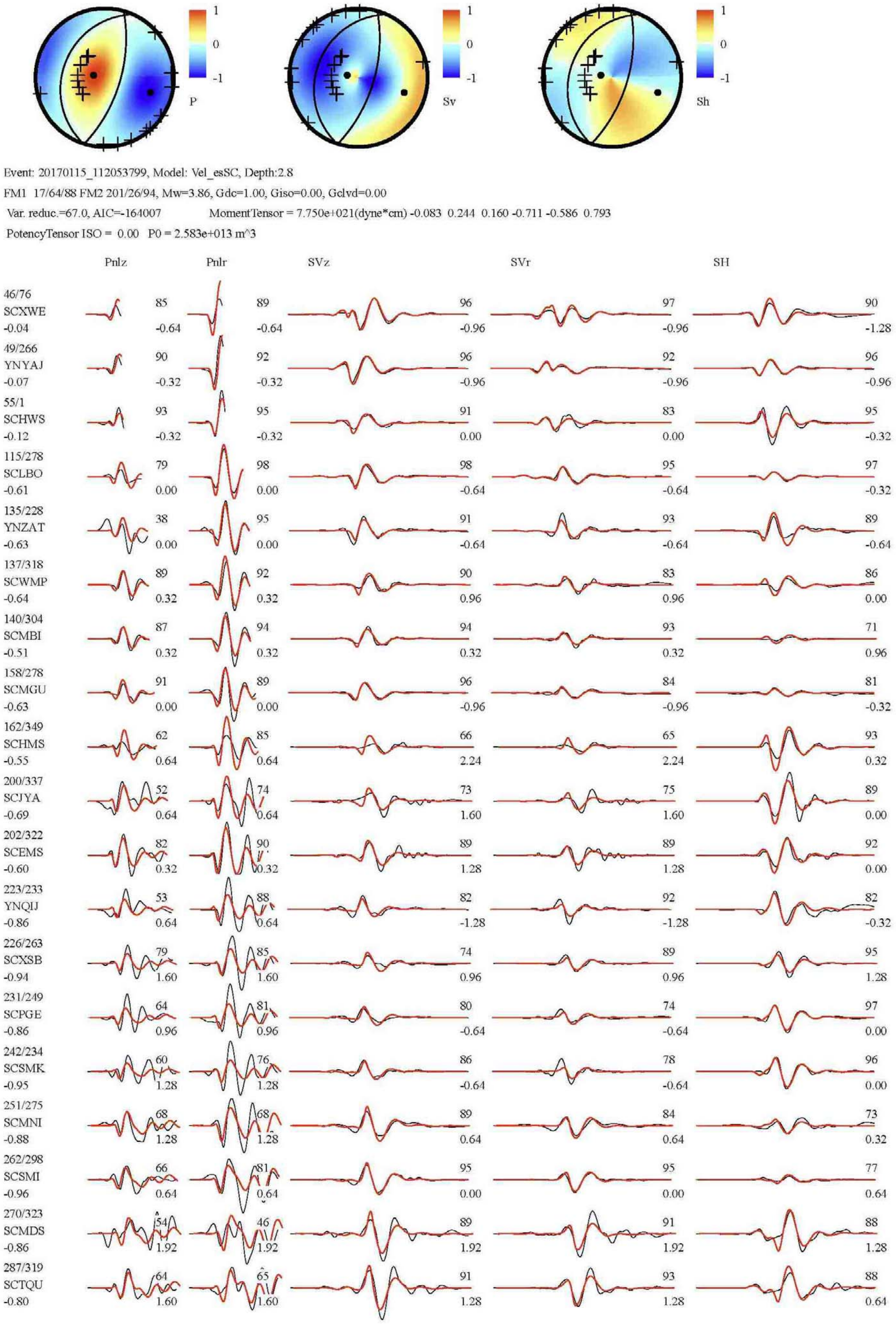

Supplementary Figure 13: Waveform matches for the 15 Jan. 2017 M_L 3.7 earthquake. See Sup. Fig. 9 caption for details.

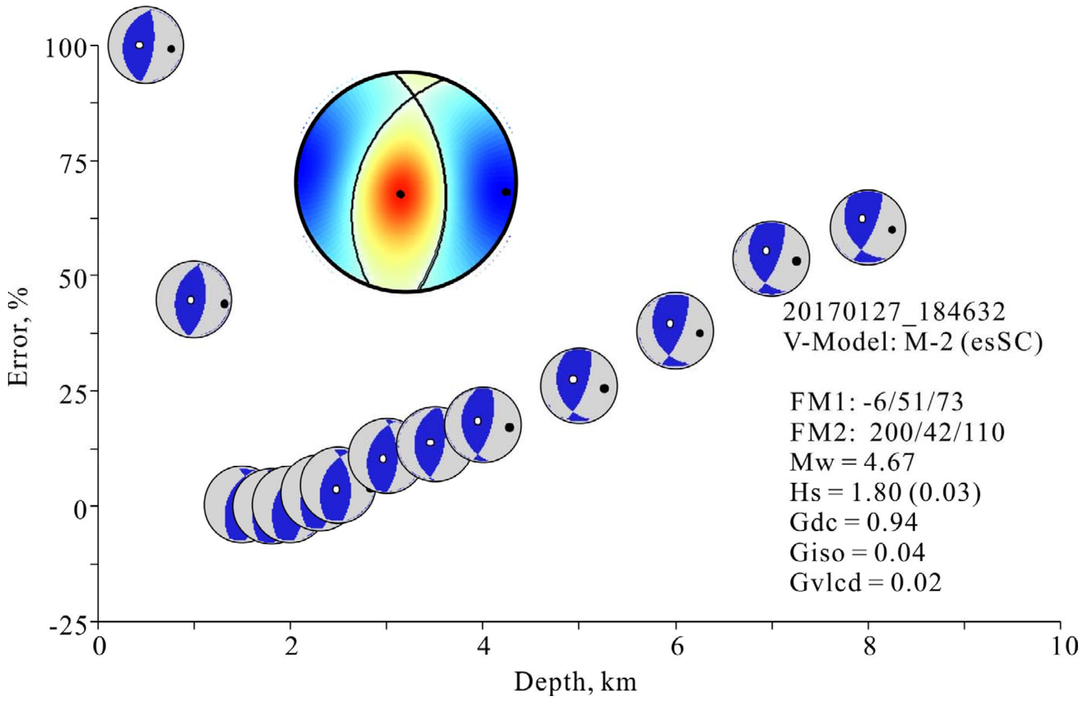

Supplementary Figure 14: Misfit error of moment tensor inversion by gCAP method against assumed focal depth for the 28 Jan. 2017 M_L 4.9 earthquake, which yielded a reverse mechanism with a strike-slip component.

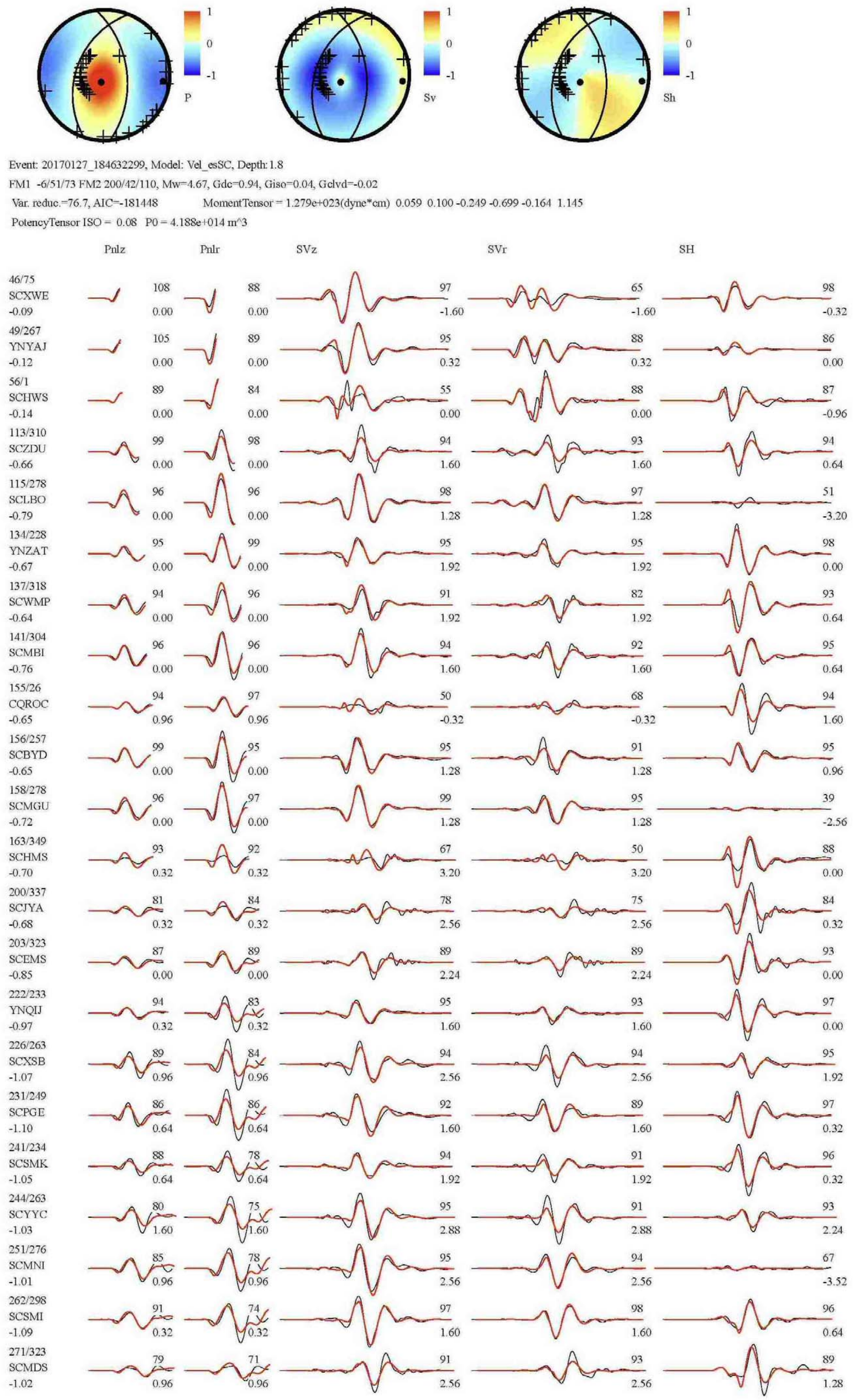

Supplementary Figure 15: Waveform matches for the 28 Jan. 2017 M_L 4.9 earthquake. See Sup. Fig. 9 caption for details.

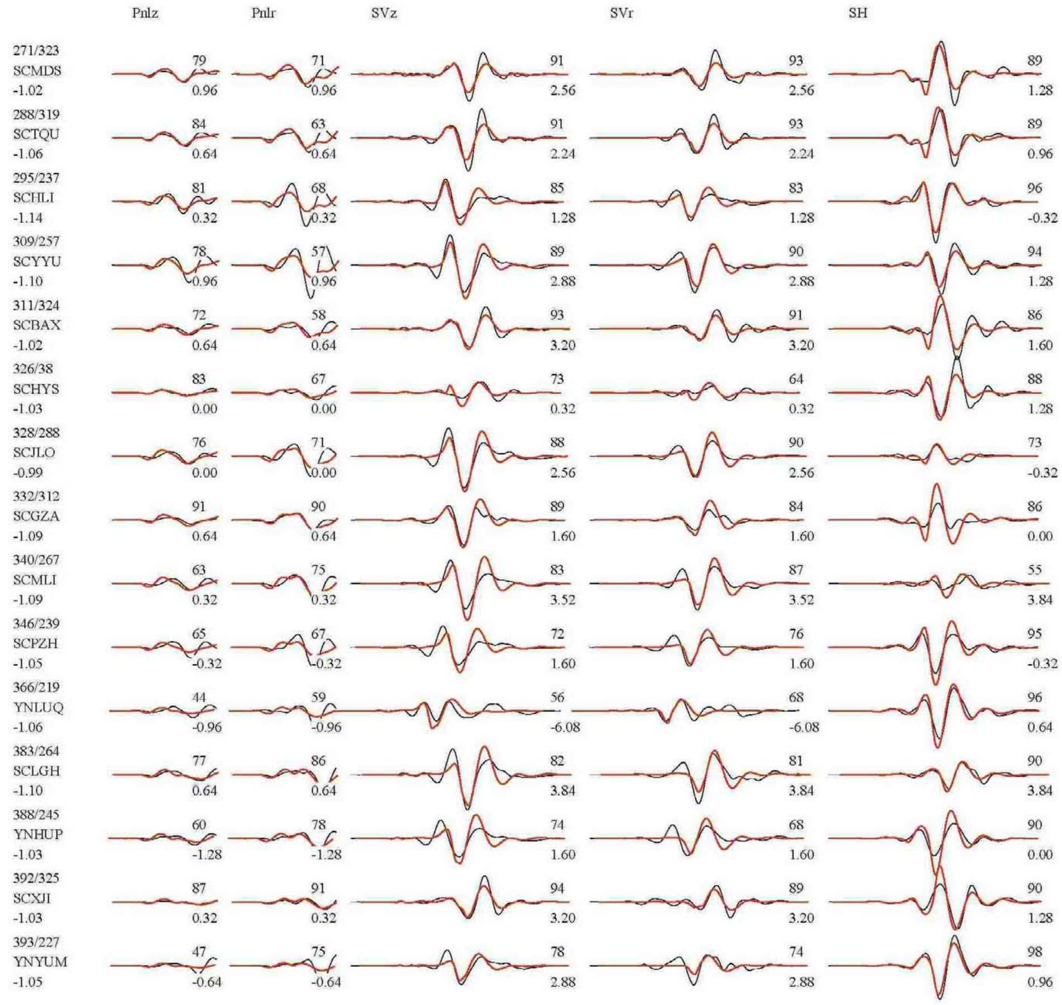

Supplementary Figure 15: Continued.

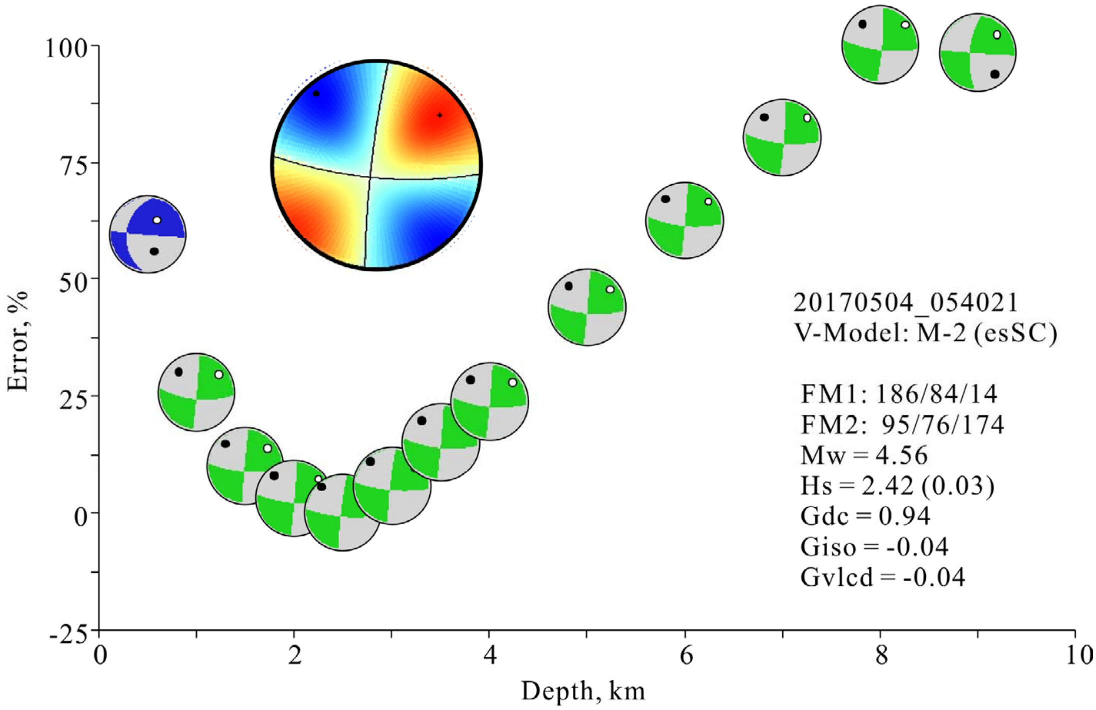

Supplementary Figure 16: Misfit error of moment tensor inversion by gCAP method against assumed focal depth for the 4 May 2017 M_L 4.9 earthquake, which yielded a reverse mechanism with a strike-slip component.

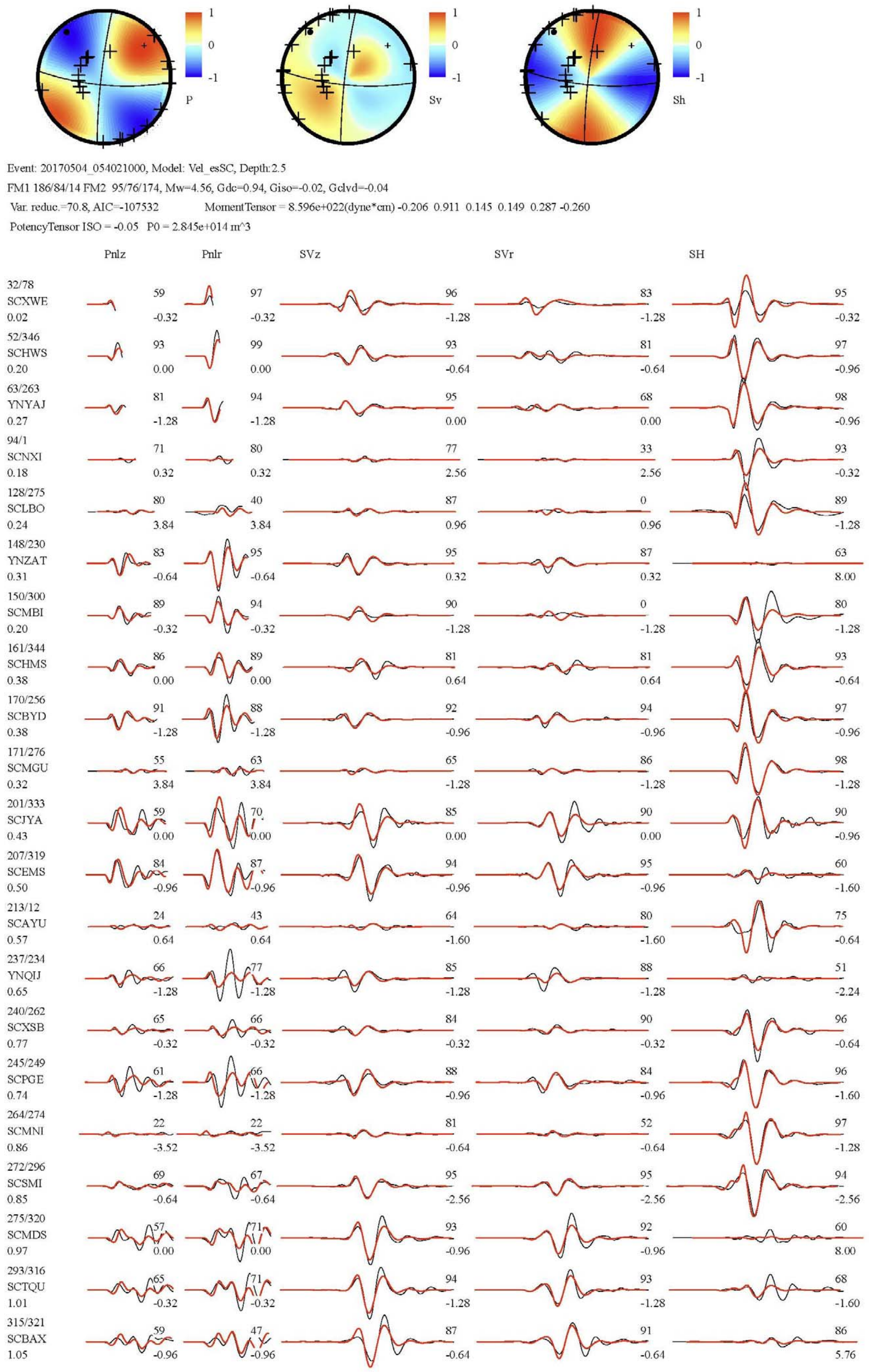

Supplementary Figure 17: Waveform matches for the 4 May 2017 M_L 4.9 earthquake. See Sup. Fig. 9 caption for details.

Supplementary Information 6: Hypocenter Relocation

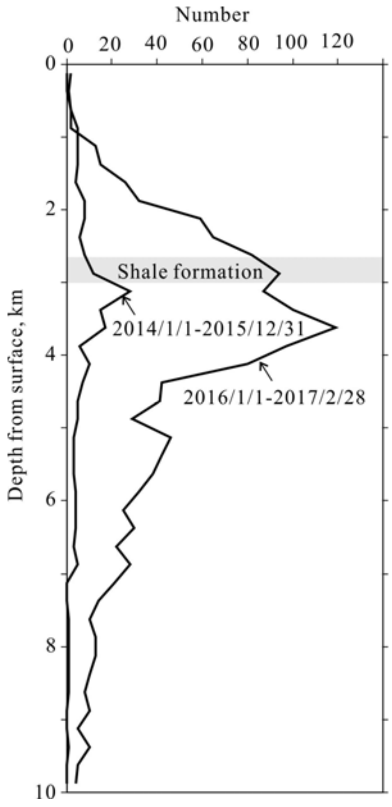

Supplementary Figure 18: Depth distribution of relocated hypocenters, showing a peak at depths of 3 to 4 km, with more events located in the upper parts of the underlying formations beneath the shale formation

Supplementary Information 7: Coulomb Failure Stress

Sup. Table 2 lists the hydraulic and mechanical properties used in the coupled thermal-hydraulic-mechanic (THM) simulation (see Methods of the main text). Sup. Fig. 19 shows Change of Coulomb failure stress (ΔCFS) distribution calculated for favorably oriented strike-slip faults of (strike, dip, rake) = (65, 90, 180). Due to the combined effect of pore pressure and solid stress, the ΔCFS shows a complex pattern. In the map view, the left and right sides (X+, X-) of the injection segment show positive ΔCFS, while the front and back regions (Y+, Y-) show negative ΔCFS stress shadows. Sup. Fig. 20 shows the results of favorably oriented reverse faults of (strike, dip, rake) = (200, 45, 90). Note the stress shadows in the underlying and overlaying formations.

The stress shadows resulting from solid deformations are located in zones where pore pressure change is minor. We also included some high permeability fault zones into the tested models so that, if such permeable channels connect with the fracturing zones, pore pressure in the fault zone could diffuse to much greater distances, up to a few kilometers away. Thus, fault zones in the stress shadow could also show positive ΔCFS. Without such permeable channels, significant pore pressure increases are limited in shale formations and restricted to within a distance of approximately 500 m.

Property / Layer	1	2	3
Bulk modulus (GPa)	9	9	9
Shear modulus (GPa)	5.4	5.4	5.4
Cohesion (MPa)	-	1	-
Tension strength (MPa)	-	20	-
Friction angle (°)	-	30	-
Dilation angle (°)	-	20	-
Biot's coefficient	0.8	0.8	0.8
Permeability (k_0) (m^2)	1×10^{-19}	$(3,3,1.8)\times10^{-17}$	1×10^{-19}
Permeability (k)	$k = k_0(1+\beta\Delta\varepsilon_v), \beta = 30000$		
Porosity	0.05	0.054	0.05

Supplementary Table 2: Major hydraulic and mechanical properties used in the coupled THM simulation.

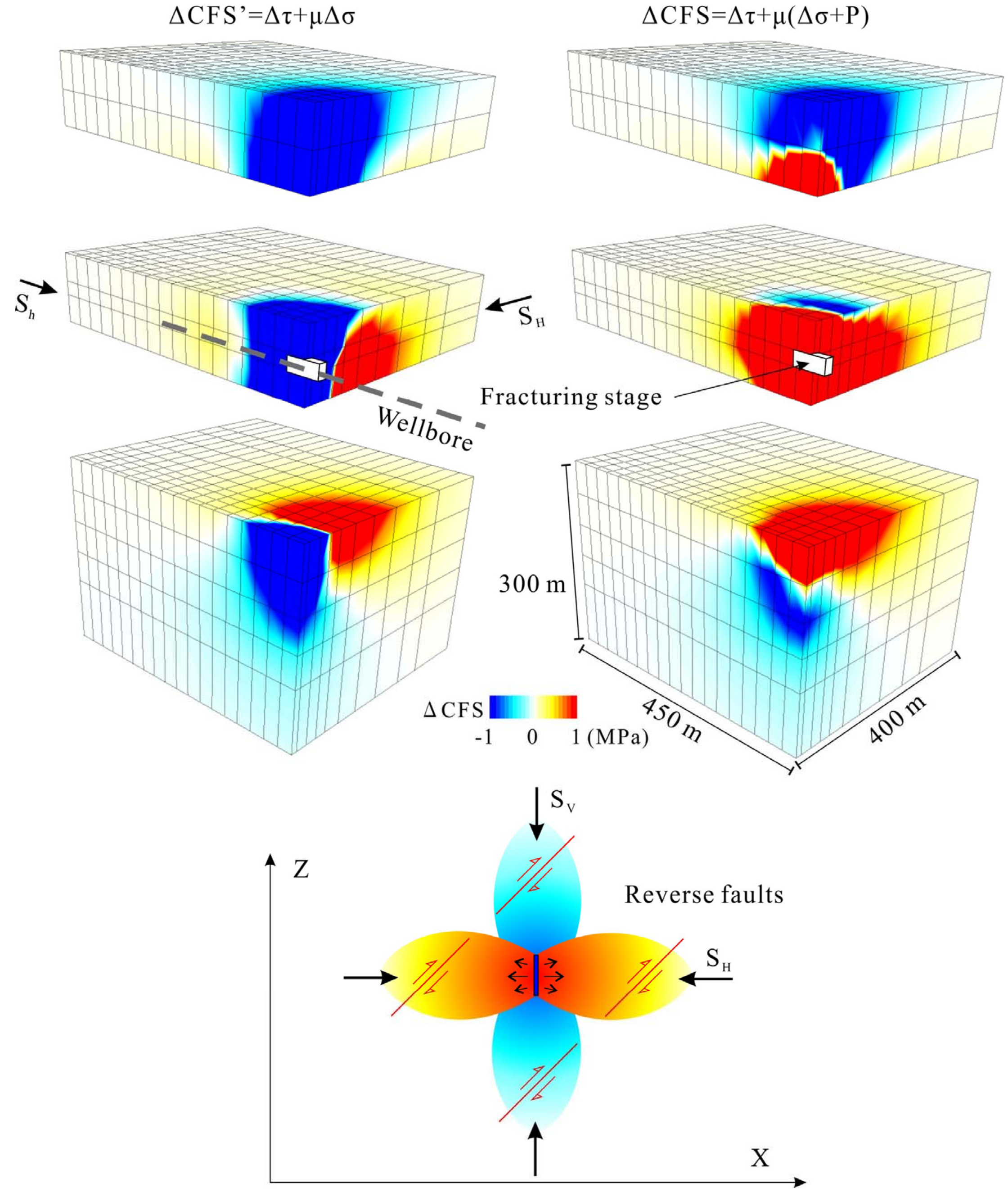

Supplementary Fig 19: Estimated ΔCFS distributions on favorably oriented strike-slip faults from a typical single stage injection. The left plots show ΔCFS by solid frame stress redistribution, while the right plot shows total ΔCFS including that from pore pressure diffusion. The lower plot is an illustration showing the ΔCFS pattern on a map view.

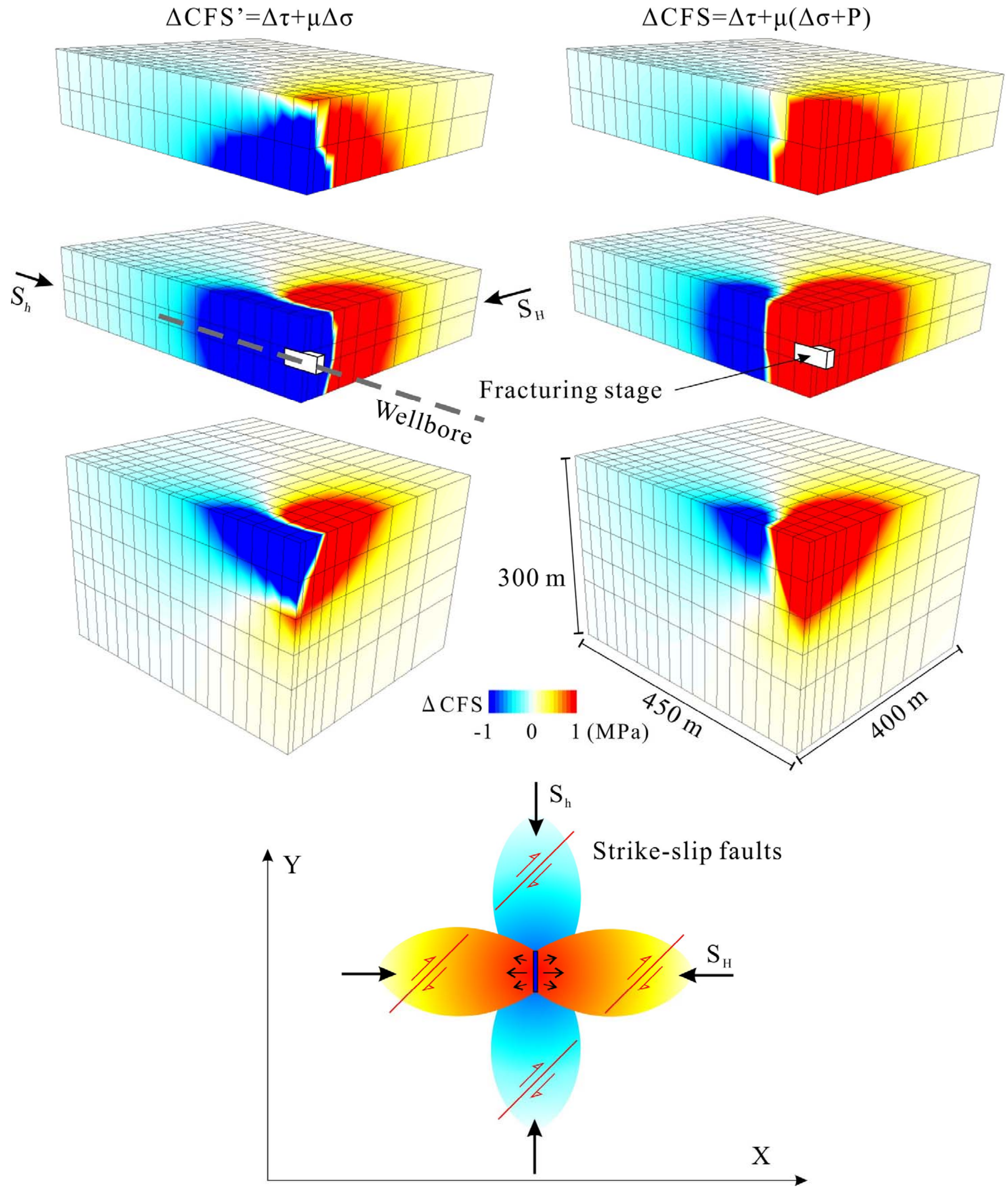

Supplementary Figure 20: Estimated distributions of ΔCFS on favorably oriented reverse faults from a typical single stage injection. The left plots show ΔCFS by solid frame stress redistribution, while the right plot shows total ΔCFS including that from pore pressure diffusion. The lower plot is an illustration showing the ΔCFS pattern on a vertical section view.

Supplementary Information 8: Damages caused by the largest events

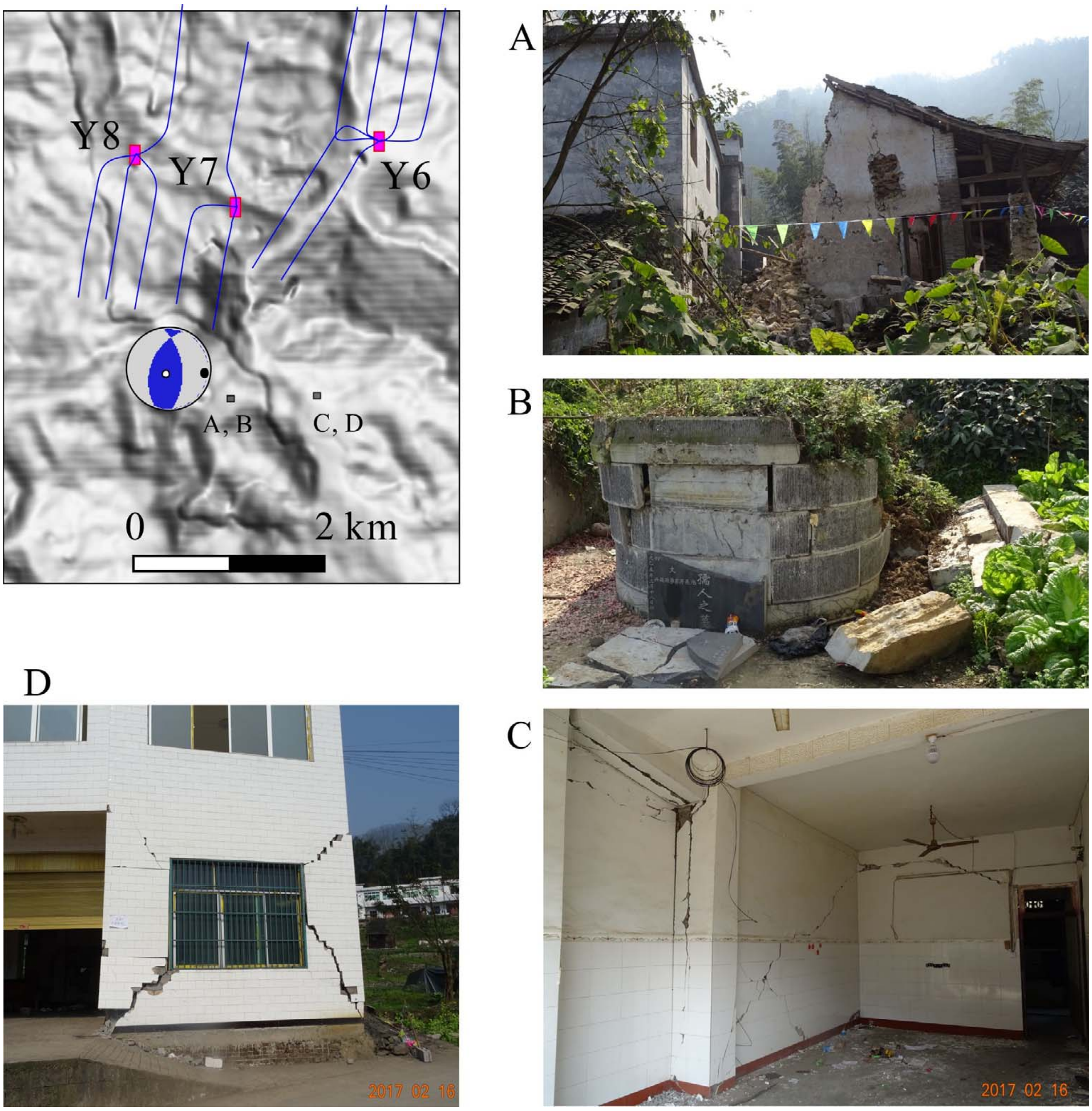

Supplementary Figure 21: Damages caused by 28 Jan. 2017 M_W 4.7 earthquake. A) A partially collapsed old house. B) Collapsed gravestones. C, D) Damages to newly built concrete houses. The map shows positions of these photos, well traces, and the location and mechanism of the earthquake. According to a formal report of the Sichuan Earthquake Administration, 23 houses collapsed and 548 houses were heavily damaged by this quake. (http://www.scdzj.gov.cn/dzpd/dzzj/ljysdzzt_2699/zqcqgg/201702/t20170203_41434.html). The index map was created using the free software GeoTaos_map (developed by Xinglin Lei; https://staff.aist.go.jp/xinglin-lei/) and finished with the software CorelDRAW X8. (Copyright (c) 2016 Corel Corporation. All rights reserved.)

Supplementary Information 9: Injection-induced seismicity at Weiyuan shale gas site

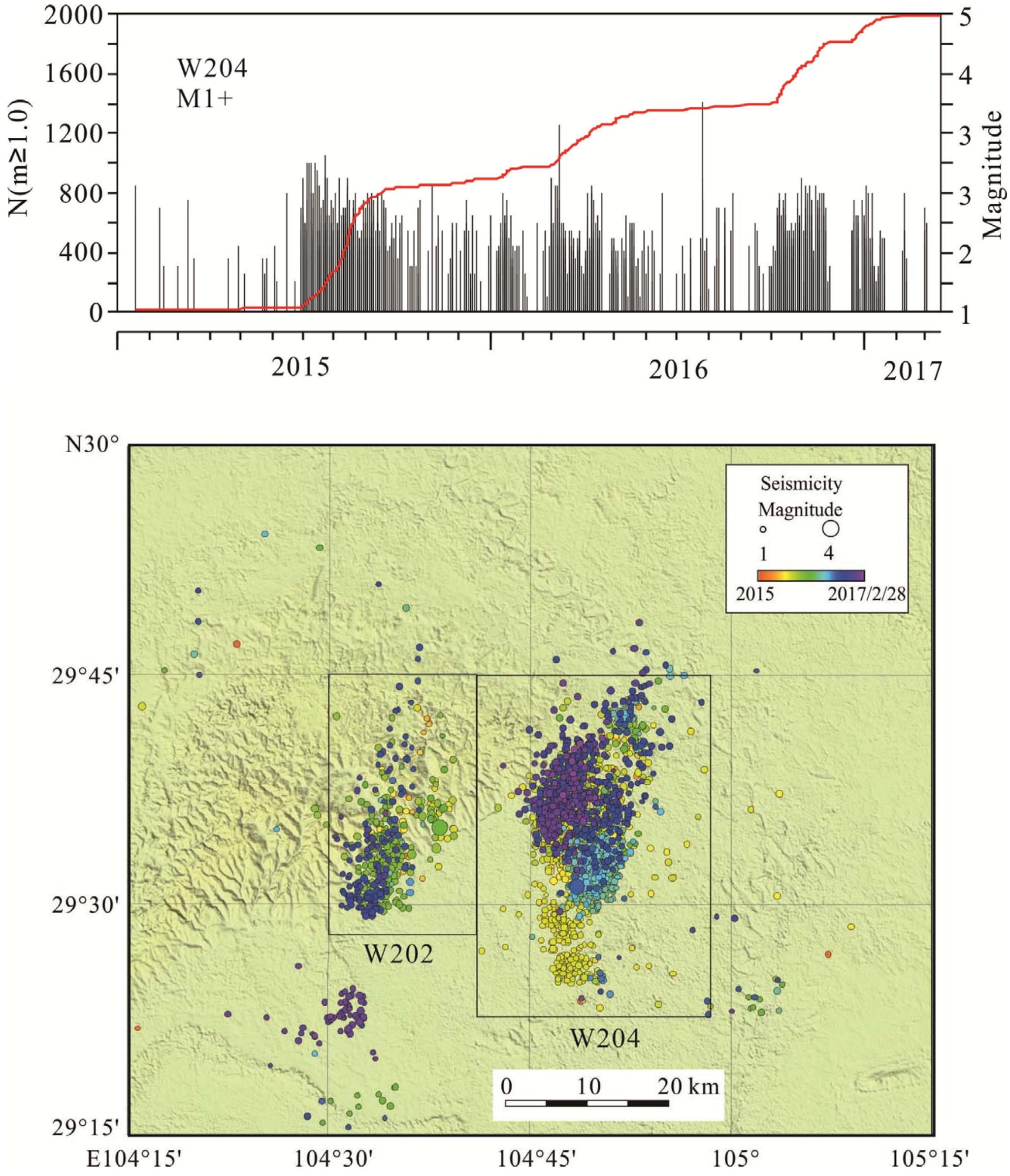

Supplementary Figure 22: Seismicity induced by hydraulic fracturing in the Weiyuan shale gas site ('B' in Fig.1 of the main text) in the central uplift of the Sichuan Basin. The top plot shows magnitude and event count of the earthquakes that occurred in W204 block, as indicated in the map plot. The lower map view shows shale blocks and location of routinely determined earthquake hypocenters. The earthquake hypocenters are scaled by magnitude and colored by date. The map was created using the free software GeoTaos_map (developed by Xinglin Lei; https://staff.aist.go.jp/xinglin-lei/) and finished with the software CorelDRAW X8. (© 2016 Corel Corporation. All rights reserved.)

REFERENCES

1 Lei, X. *et al.* A detailed view of the injection-induced seismicity in a natural gas reservoir in Zigong, southwestern Sichuan Basin, China. *Journal of Geophysical Research: Solid Earth* **118**, 4296-4311, doi:10.1002/jgrb.50310 (2013).

2 Lei, X., Yu, G., Ma, S., Wen, X. & Wang, Q. Earthquakes induced by water injection at ~3 km depth within the Rongchang gas field, Chongqing, China. *Journal of Geophysical Research* **113**, doi:10.1029/2008jb005604 (2008).

3 Zou, C. N. *et al.* Conditions of shale gas accumlation and exploration practices in China. *Natural Gas Industry. (in Chinese with English abstract)* **31**, 26-39 (2011).

4 Jia, C., Zheng, M. & Zhang, Y. Unconventional hydrocarbon resources in China and the prospect of exploration and development. *Petroleum Exploration and Development* **39**, 139-146 (2012).

5 EIA. *Technically recoverable shale oil and shale gas resources: China.* (U.S. Department of Energy, 2013).

6 Xin-gang, Z. & Ya-hui, Y. The current situation of shale gas in Sichuan, China. *Renewable and Sustainable Energy Reviews* **50**, 653-664, doi:10.1016/j.rser.2015.05.023 (2015).

7 Ruan, X., Cheng, W. Z., Zhang, Y. J., Li, J. & Chen, Y. Research of the earthquakes induced by water injections in salt mines in Changning. *Earthquake Research in China. (in Chinese)* **24**, 226-234 (2008).

8 Wang, X. *et al.* S-wave velocity of the crust in Three Gorges Reservoir and the adjacent region inverted from seismic ambient noise tomography. *Chinese J. Geophys. (in Chinese with English abstract)* **56**, 1-11, doi:10.6038/cjg20131201 (2013).

9 CNPC, R. I. o. S. a. E. P. T. Environmental Impact Statement of CNPC Changning, Weiyuan, and Zhaotong shale gas construction project (in Chinese). 381 (2015).

10 Zhu, L. & Ben-Zion, Y. Parametrization of general seismic potency and moment tensors for source inversion of seismic waveform data. *Geophysical Journal International* **194**, 839-843 (2013).
